# Studying Microtemporal, Within-Person Processes of Diet, Physical Activity, and Related Factors Using the APPetite-Mobile-App: Feasibility, Usability, and Validation Study

**DOI:** 10.2196/25850

**Published:** 2021-07-05

**Authors:** Alea Ruf, Elena Doris Koch, Ulrich Ebner-Priemer, Monika Knopf, Andreas Reif, Silke Matura

**Affiliations:** 1 Department of Psychiatry, Psychosomatic Medicine and Psychotherapy University Hospital Goethe University Frankfurt Germany; 2 Mental mHealth Lab Institute of Sports and Sports Science Karlsruhe Institute of Technology Karlsruhe Germany; 3 Department of Psychiatry and Psychotherapy Central Institute of Mental Health, Medical Faculty Mannheim Heidelberg University Mannheim Germany; 4 Department of Developmental Psychology Goethe University Frankfurt Germany

**Keywords:** diet, physical activity, microtemporal processes, within-person factors, ecological momentary assessment, smartphone-app, mobile phone, mHealth, dietary assessment, feasibility, usability, validity

## Abstract

**Background:**

Diet and physical activity (PA) have a major impact on physical and mental health. However, there is a lack of effective strategies for sustaining these health-protective behaviors. A shift to a microtemporal, within-person approach is needed to capture dynamic processes underlying eating behavior and PA, as they change rapidly across minutes or hours and differ among individuals. However, a tool that captures these microtemporal, within-person processes in daily life is currently not present.

**Objective:**

The APPetite-mobile-app is developed for the ecological momentary assessment of microtemporal, within-person processes of complex dietary intake, objectively recorded PA, and related factors. This study aims to evaluate the feasibility and usability of the APPetite-mobile-app and the validity of the incorporated APPetite-food record.

**Methods:**

The APPetite-mobile-app captures dietary intake event-contingently through a food record, captures PA continuously through accelerometers, and captures related factors (eg, stress) signal-contingently through 8 prompts per day. Empirical data on feasibility (n=157), usability (n=84), and validity (n=44) were collected within the Eat2beNICE-APPetite-study. Feasibility and usability were examined in healthy participants and psychiatric patients. The relative validity of the APPetite-food record was assessed with a subgroup of healthy participants by using a counterbalanced crossover design. The reference method was a 24-hour recall. In addition, the energy intake was compared with the total energy expenditure estimated from accelerometry.

**Results:**

Good feasibility, with compliance rates above 80% for prompts and the accelerometer, as well as reasonable average response and recording durations (prompt: 2.04 min; food record per day: 17.66 min) and latencies (prompts: 3.16 min; food record: 58.35 min) were found. Usability was rated as moderate, with a score of 61.9 of 100 on the System Usability Scale. The evaluation of validity identified large differences in energy and macronutrient intake between the two methods at the group and individual levels. The APPetite-food record captured higher dietary intakes, indicating a lower level of underreporting, compared with the 24-hour recall. Energy intake was assessed fairly accurately by the APPetite-food record at the group level on 2 of 3 days when compared with total energy expenditure. The comparison with mean total energy expenditure (2417.8 kcal, SD 410) showed that the 24-hour recall (1909.2 kcal, SD 478.8) underestimated habitual energy intake to a larger degree than the APPetite-food record (2146.4 kcal, SD 574.5).

**Conclusions:**

The APPetite-mobile-app is a promising tool for capturing microtemporal, within-person processes of diet, PA, and related factors in real time or near real time and is, to the best of our knowledge, the first of its kind. First evidence supports the good feasibility and moderate usability of the APPetite-mobile-app and the validity of the APPetite-food record. Future findings in this context will build the foundation for the development of personalized lifestyle modification interventions, such as just-in-time adaptive interventions.

## Introduction

### Background

Diet is a key contributor to both physical and mental health. Elevated BMI is a major risk factor for noncommunicable diseases, such as cardiovascular diseases [1]. Since 1975, the prevalence of obesity has nearly tripled globally [1]. Accordingly, in 2016, 13% of adults were obese and 39% were overweight [1]. Approximately 11 million deaths were associated with dietary risk factors (eg, low intake of whole grains) across 195 countries in 2017 [2]. Although the link between diet and mental health is not equally well understood, first evidence supports the presence of a direct association among diet, mental health, and mental functioning [3]. Obesity not only increases the probability of somatic diseases but also of mental illness, particularly depression [4-6]. These numbers and findings highlight the growing need to understand the “causes of the causes.”

Although factors and processes underlying eating behavior have been studied for many years [7,8], interventions remain ineffective in sustaining health-protective behaviors for the long term [9]. One reason for this could be the main focus on between-person characteristics (eg, age) and macrotemporal processes (across weeks, months, or years) [10]. Diet is a highly complex health behavior that is performed multiple times per day and is influenced by a variety of fluctuating factors and their interactions [11]. A real-life microtimescale approach is needed to capture the dynamics of diet and associated factors ecologically and momentarily and, ultimately, to understand the processes underlying eating behavior in everyday life [10]. In contrast to some between-person characteristics (eg, age), within-person factors are modifiable and therefore a promising target for interventions. For this reason, the identification of within-person factors that influence eating behavior in daily life is needed for the development of novel, more effective, and personalized interventional approaches.

It is not only diet that has a large impact on both physical and mental health. Physical activity (PA) represents another impactful, repeated-occurrence health behavior [12,13]. To untangle the complex association between diet and health [14], it is important to consider possible interactions. For instance, diet does not independently regulate body weight. Body weight is regulated through the interplay of energy intake (ie, diet) and energy expenditure (eg, PA) [15]. Therefore, the assessment of microtemporal, within-person processes of diet and PA should be combined, and possible interactions should be taken into consideration.

### Ecological Momentary Assessment of Diet, PA, and Related Factors

The repeated or continuous assessment of experiences, behaviors, or physiological processes in real life through smartphones or wearable devices is a highly promising approach for studying microtemporal, within-person processes [16]. This approach is referred to as ecological momentary assessment (EMA), ambulatory assessment, experience sampling, and real-time data capture [17]. Although different terms have been used, they have in common the assessment of various parameters, multiple times per day in daily life [17].

Even though EMA studies do not allow causal conclusions, they offer insight into three important aspects of microtemporal, within-person processes: (1) temporal specificity (eg, Does diet influence mood to a greater extent than mood influences diet?), (2) situational specificity (eg, Is unhealthy eating more likely when being alone or with others?), and (3) person specificity (eg, Is stress more predictive for engaging in eating for some individuals compared with others?) [10].

Diet is a highly complex phenomenon that makes its assessment difficult. However, to avoid typical reporting biases that are present in traditional dietary assessment methods (eg, food frequency questionnaires), the number of studies using EMA to capture self-reported dietary intake or aspects of it in real time or near real time instead of retrospectively has rapidly grown in the last decade [18-20]. There are two categories of EMA approaches present so far: on the one hand, there are mobile-based dietary assessment tools that focus on the assessment of complex dietary intake and the generation of nutritional values. Complex dietary intake refers to assessing all consumed foods and drinks and consumed amounts, which are then used to generate nutritional values. Even though a small number of tools that assess complex dietary intake also allow assessing contextual correlates during eating occasions [21], no tool allows capturing a wider repertoire of factors preceding or succeeding eating occasions [22]. On the other hand, there are a number of studies that use EMA to study a variety of factors related to diet (eg, affect [23]). However, to the best of our knowledge, none of these studies assessed diet in its full complexity. Most of them focus on specific aspects of diet only, for example, snacks or sweetened beverages [24-29], a limited number of food and drink categories [23,30-33], portion sizes [34], or the type of eating events (main meals vs snacks) and the type of drinking occasions (alcoholic vs nonalcoholic) [35]. Hence, complex dietary intake was not assessed, and the generation of nutritional values was not possible. Although some of these studies reported a more comprehensive approach which captured all consumed foods and, in some studies, drinks through a free input field [23,33], the foods and drinks were only assigned to a limited number of food and drink categories and were not used to generate nutritional values. There is a need to study the processes underlying complex dietary intake instead of processes underlying only aspects of diet.

Despite the importance of taking possible interactions into account, most EMA studies focus on either the assessment of diet or PA. One study identified the need for an EMA tool to capture *complex lifestyle behavior*, that is, dietary intake and PA simultaneously [36]. However, the tool developed for this purpose failed to assess diet and PA in their complex nature. It only assessed specific food categories and used self-reports for the assessment of PA, which is unsatisfactory, given that 2 systematic reviews showed that indirect measures of PA (ie, self-reports) differ substantially from direct, objective measures (eg, accelerometers) [37,38].

In conclusion, there is a strong need for an EMA tool that allows capturing complex dietary intake, objectively measured PA, and a broad range of associated factors simultaneously in daily life to study microtemporal, within-person processes underlying these health-protective behaviors.

### Objectives

As no EMA tool allows the study of microtemporal, within-person processes of complex dietary intake, objectively measured PA, and related factors, we developed an EMA tool for the simultaneous assessment of these complex health behaviors and related factors in daily life: the APPetite-mobile-app (this term also covers the assessment of PA, although it is not performed by the APPetite-mobile-app itself but by an accelerometer).

The suitability of novel EMA tools for use in daily life should be evaluated. Therefore, feasibility, usability, and validity were examined empirically in this study. The following questions will be addressed: Is the APPetite-mobile-app a feasible and usable tool for the combined assessment of complex dietary intake, PA, and associated factors in daily life and a valid tool for the assessment of complex dietary intake in real time or near real time?

## Methods

### The APPetite-Mobile-App

#### Software and Hardware

The APPetite-mobile-app was developed and run through movisensXS (version 1.4.7, movisens GmbH), a web-based platform for the development of EMA tools. It supports a broad range of sampling schemes, item formats, and multimedia records, allowing flexible and tailored study configurations. The APPetite-mobile-app is run through the movisensXS app (available for Android devices). If the mobile device has access to mobile data during the EMA assessment, participants’ entries will be uploaded instantly to the platform. In this way, compliance can be monitored throughout the EMA assessment, and a chat function allows direct messaging with participants. All participants received a study smartphone (Motorola Moto G 3rd generation), with access to mobile data. The movisensXS app was previously tested on this particular mobile device, ensuring its smooth functioning and increasing the standardization of the mobile-based assessment.

#### Sampling Strategy

**Figure 1:**
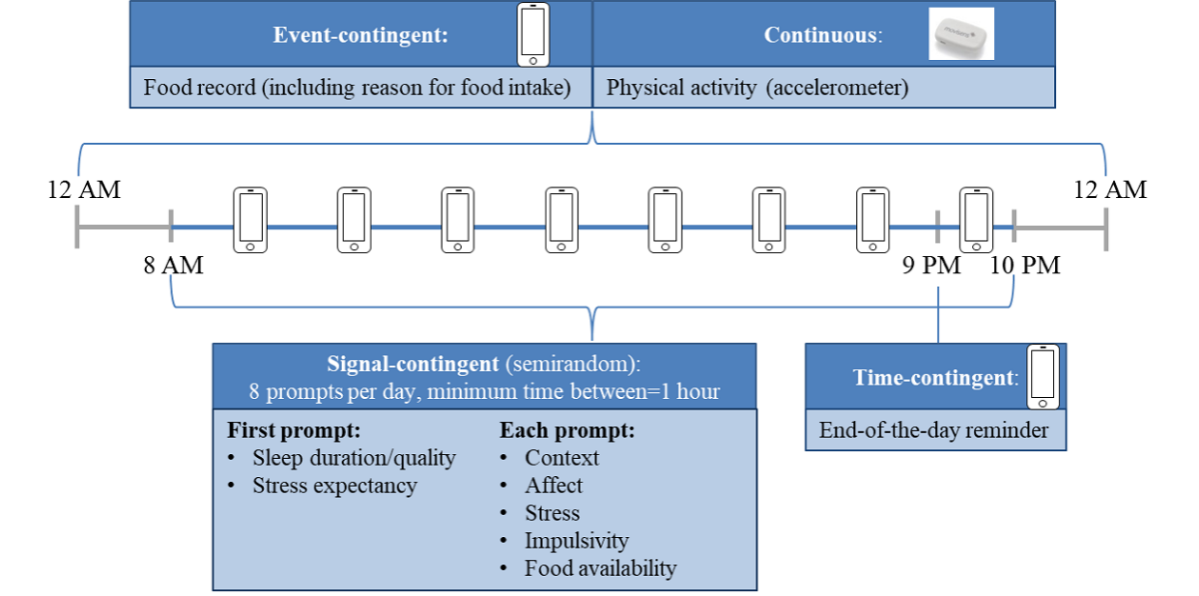
Sampling strategy of the APPetite-mobile-app.

The APPetite-mobile-app uses event-, signal-, and time-contingent as well as continuous sampling (Figure 1).

Food intake was recorded event-contingently through a food record. Participants were asked to enter foods and drinks as soon as possible after consuming them. Accordingly, participants are able to initiate the APPetite-food record at any time and capture their food intake in real time. This was chosen to minimize memory effects and record the exact time of food intake. In addition, this allows capturing food intake even during the night, when signal-contingent prompts are inappropriate. At 9 PM, a time-contingent prompt asks if all consumed foods and drinks of the day have been recorded, ensuring that no foods and drinks consumed on this day are missed.

The prompts are initiated signal-contingent at eight semirandom times per day between 8 AM and 10 PM. The minimum time between 2 prompts is 1 hour. Therefore, participants cannot predict the exact time of the next prompt, and the assessed situation is a better reflection of the participant’s real life. Participants were instructed to respond immediately to the prompt. However, if participants are unable to reply instantly, it is possible to postpone the prompt for 5, 10, 15, 20, or 25 minutes to avoid missing data and reduce the participants’ burden. If no reaction is registered, the prompt is deactivated and cannot be reactivated.

Continuous sampling through an accelerometer is used for the assessment of PA.

#### EMA Measures

##### APPetite-Food Record

**Figure 2:**
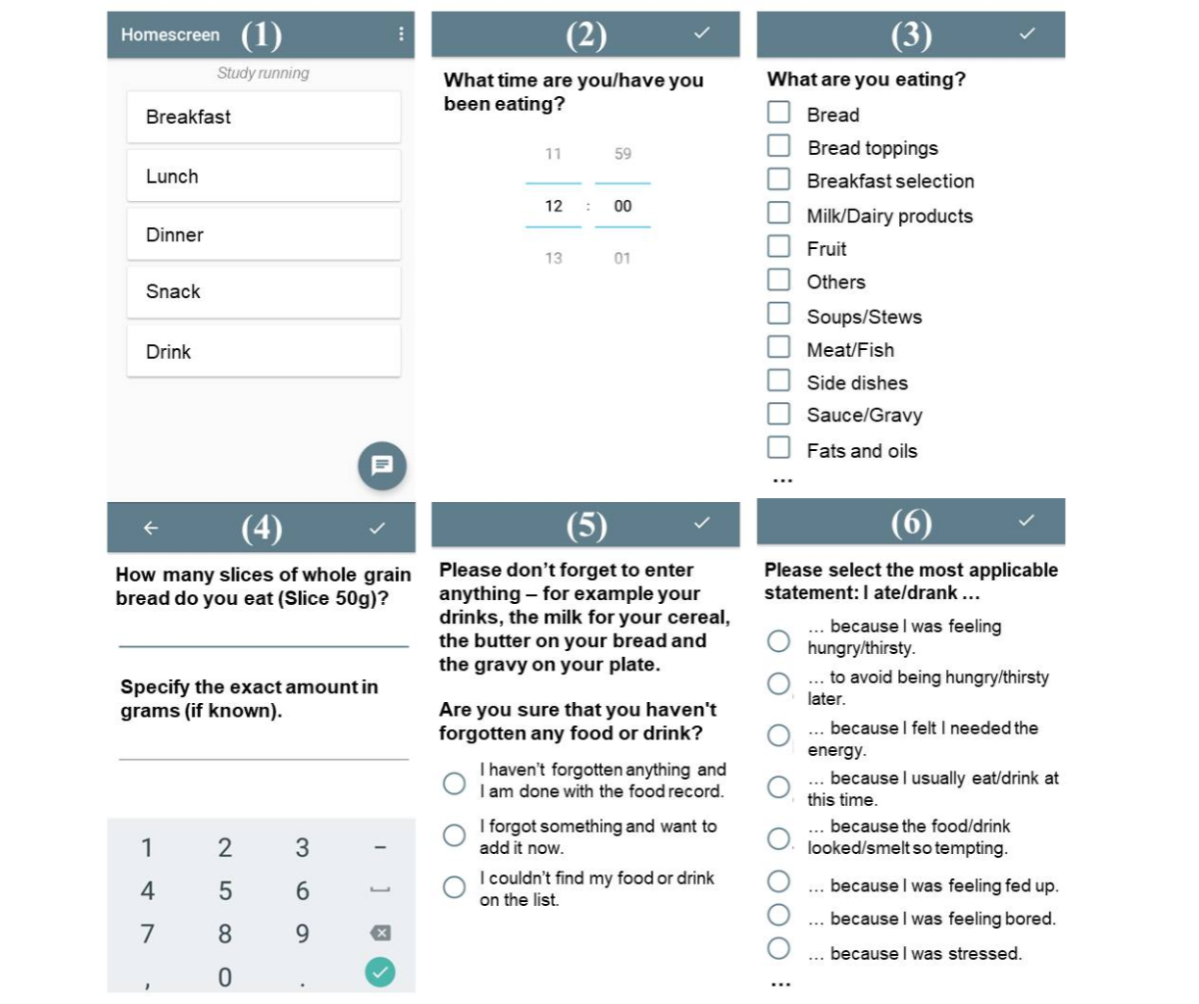
Screenshots of the 6-step process of the APPetite-food record.

The APPetite-food record comprises a 6-step process: (1) selection of meal type, (2) entry of time of intake, (3) selection of consumed foods and drinks, (4) specification of consumed amounts, (5) presentation of reminder for commonly forgotten foods, and (6) indication of predominant reason for eating or drinking (Figure 2 presents screenshots of the 6-step process). To generate nutritional values, the obtained dietary data were transferred by trained staff to myfood24-Germany, a 24-hour dietary recall [39]. A detailed description of the APPetite-food record and nutritional value generation is provided in Multimedia Appendix 1 [39-44]. All reasons for eating and drinking are presented in Multimedia Appendix 2 [40-44].

##### Prompts

Each prompt assesses the context, affect, stress, impulsivity, and food availability either since the last prompt or immediately before the prompt. In addition, the first prompt of a day captures sleep quality and quantity as well as stress expectancy. All prompt measures and items are described in Multimedia Appendix 3 [45-49].

##### Physical Activity

Move 3 sensors from movisens were used to objectively record PA. The accelerometer was worn on the nondominant wrist. Participants were asked to wear it at any time (also when sleeping) and only take it off when showering or performing water activities. The Move 3 sensor captures raw data on 3D acceleration, barometric air pressure, and temperature. Secondary parameters such as activity class, body position, steps, metabolic equivalents, and PA metrics can be extracted using the DataAnalyzer (movisens GmbH).

### Evaluation of Feasibility

#### Measures

The feasibility of the APPetite-mobile-app was separately assessed for the EMA prompts, the APPetite-food record, and the accelerometer. The feasibility of the prompts is determined by prompt delivery, total number of answered prompts across all subjects, number of answered prompts per participant, compliance (percentage of complete prompts within received prompts), response latency (time from first prompt signal to answering), and the time needed to complete a single prompt. The food record’s feasibility was evaluated based on the number of recorded eating and drinking events per day, reporting latency (time between the meal and meal recording), and the time needed to record food intake per day. The amount of time wearing the accelerometer and compliance (percentage wearing the accelerometer within the 7-day assessment period) are measures of feasibility of the accelerometer.

#### Sample

The data were collected within the ongoing APPetite study. The APPetite study is part of the European Union Horizon2020 project Eat2beNICE and recruits participants from three existing studies: LORA (Longitudinal Resilience Assessment) study [50], PROUD (Prevention of Comorbid Depression and Obesity in Attention-Deficit/Hyperactivity Disorder) study [51], and the BipoLife-A1 study (improving early recognition and intervention in people at risk of developing bipolar disorder [52,53]). The LORA study included individuals who were not affected by psychiatric conditions. The PROUD sample consisted of patients affected by attention-deficit/hyperactivity disorder. The BipoLife-A1 study follows up on patients with an increased risk for the development of bipolar disorder, including patients affected by attention-deficit/hyperactivity disorder or depression.

From November 2018 to March 2020, 161 participants were included in the APPetite study (140 LORA, 7 PROUD, and 14 BipoLife-A1). After the first in-person session, 3 LORA participants dropped out. Of these, 2 realized that they were unable to respond to prompts. The third person was mistakenly given a smartphone that was not coupled with the EMA protocol. Another person dropped out after the additional in-person session of the validation study for private reasons. Hence, EMA data of 157 participants are available for the evaluation of feasibility (see demographics in Table 1).

**Table 1:** Demographics of the total sample and the 3 cohorts (only individuals who completed the ecological momentary assessment were included; N=157).

Variables		Total (N=157)	LORA^a^ (n=136)	PROUD^b^ (n=7)	BipoLife-A1 (n=14)
**Gender, n (%)**					
	Female	100 (63.7)	94 (69.1)	1 (14.3)	5 (35.7)
	Male	57 (36.3)	42 (30.9)	6 (85.7)	9 (64.3)
Age (years), mean (SD)		28.04 (7.22)	28.08 (7.55)	26.43 (2.51)	28.5 (5.39)
BMI, mean (SD)		24.71 (4.81)	24.26 (3.87)	24.98 (6.11)	28.9 (9.13)

^a^LORA: Longitudinal Resilience Assessment.

^b^PROUD: Prevention of Comorbid Depression and Obesity in Attention-Deficit/Hyperactivity Disorder.

#### Procedure

The APPetite study consists of 2 in-person sessions, the EMA assessment, and a follow-up session from home. In the first in-person session, participants received detailed training on how to use the APPetite-mobile-app and the accelerometer. Participants received a smartphone with the APPetite-mobile-app and an accelerometer, including a wristband. Participants used the APPetite-mobile-app for 3 consecutive days (2 weekdays and 1 weekend day, not including the day of the first in-person session) and wore the accelerometer for 7 consecutive days (overlapping the 3 days of the APPetite-mobile-app assessment, not including the day of the first in-person session). During the 3 days of the app-based assessment, prompt compliance was tracked. If compliance fell below the threshold of 80%, a motivational message was sent to the participant. Participants who completed at least 80% (19/24) of the prompts were included in a raffle to win a €100 (US $121.74) voucher and a cooking class. Before the second in-person session, EMA data were checked, and questions regarding implausible prompt entries (eg, 8 AM as bedtime) and food records (eg, missing meals) were collected. These questions were reviewed in the second in-person session to resolve any uncertainties. Usability of the APPetite-mobile-app, reactivity, and representativity of the EMA assessment were assessed via questionnaires in the second in-person session.

Participants received €40 (US $48.7) after the second in-person session and €10 (US $12.17) after completing the follow-up. In addition, individual feedback on diet and PA was provided after the follow-up, which consisted of a web-based 24-hour recall from home.

#### Statistical Methods

Descriptive statistics were used to assess feasibility measures. We investigated whether compliance differed among the 3 cohorts, the 3 days, and between male and female participants. As compliance is not normally distributed, this is done using the following nonparametric tests: Kruskal-Wallis rank sum test, Friedman test, and Wilcoxon rank sum test. In addition, the Spearman rank correlation coefficient was calculated to investigate the association between compliance and age. The α level was set to .05. The analyses were performed using R 3.6.1 (R Core Team) with RStudio (RStudio, Public-benefit corporation).

### Evaluation of Usability

#### Measures

Usability is assessed using the System Usability Scale (SUS; [54]), a commonly used questionnaire for the evaluation of websites or mobile apps. The questionnaire consists of 10 items. Each item represents a statement (eg, I thought the system was easy to use). Participants’ agreement with the statement was rated on a 5-point scale. A total score between 0 and 100 was calculated. Higher numbers indicated better usability.

#### Sample

Data were collected within the APPetite study. However, SUS was subsequently added to this study (August 2019). Therefore, it is available only for a subsample of 84 participants (55 women and 29 men; 67 from LORA, 6 from PROUD, and 11 from BipoLife-A1). The mean age of the sample was 29.26 (SD 7.41) years, and the mean BMI was 24.82 (SD 5.26) kg/m^2^.

#### Procedure

The SUS was completed during the second in-person session.

#### Statistical Methods

Total usability scores were calculated according to the study by Brooke [54] and presented through descriptive statistics (mean, SD, and range). We investigated whether usability was rated differently by the 3 cohorts using a one-way analysis of variance, as data are normally distributed and homogeneity of variance is given. An unpaired *t* test (two-tailed) was used to study gender differences, as assumptions of normal distribution and homogeneity of variance were met. The associations between usability and age (not normally distributed) as well as usability and compliance (not normally distributed) were investigated using Spearman rank correlations. The data were analyzed using R 3.6.1 with RStudio. The α level was set to .05.

### Evaluation of Validity

#### Measures

The relative validity was assessed using a counterbalanced crossover design. Myfood24 Germany (Measure Your Food on One Day), a 24-hour recall, was chosen as the reference method. Myfood24 is a web-based, self-administered 24-hour dietary recall tool (refer to Koch et al [39] for details). It is based on two German nutritional databases (the German Food Code and Nutrient Data Base, Bundeslebensmittelschlüssel version 3.02, and the database LEBTAB of the Dortmund Nutritional and Anthropometric Longitudinally Designed study) and includes 11,501 food items. A comparison between habitual energy and macronutrient intake assessed through the APPetite-food record and 24-hour recall was drawn. Habitual intake was operationalized as the mean dietary intake of 3 days.

Furthermore, energy intake is compared with total energy expenditure (TEE) based on the assumption that energy intake equals TEE in weight-stable individuals [55]. TEE is estimated from nondominant wrist accelerometry according to White et al [56], which has been shown to be a precise approach to estimate TEE on population levels in free-living conditions when compared with TEE by doubly labeled water. The Euclidean norm minus one was extracted from the raw acceleration data using the DataAnalyzer from movisens (version 1.13.5; June 18, 2019) and inserted into the quadratic Euclidean norm minus one equation from White et al [56].

#### Sample

A total of 50 healthy participants from the LORA study (group 1: n=26; group 2: n=24) volunteered for the validation study. However, 6 participants from group 1 had to be excluded, as they did not complete all relevant parts within the predefined time schedule. Therefore, the evaluation of validity was based on data from 44 participants (33 women and 11 men)—20 from group 1 and 24 from group 2. This sample had a mean age of 28.64 (SD 8.13) years and a mean BMI of 23.8 (SD 3.62) kg/m^2^. The groups did not significantly differ in terms of sex (group 1: 15 women and 5 men; group 2: 18 women and 6 men; *X^2^*_1_=0; *P*=.99), age (group 1: mean 30.15, SD 8.65 years; group 2: mean 27.38, SD 7.63 years; Mann-Whitney *U*=314; *P*=.08), and BMI (group 1: mean 23.99, SD 3.69 kg/m^2^; group 2: mean 23.66, SD 3.63 kg/m^2^; t_40.34_=0.3 [unpaired; two-tailed]; *P*=.77).

#### Procedure

Participants from the LORA cohort who agreed to participate in the APPetite study were asked whether they wanted to also participate in the validation study: recording their food intake through a 24-hour recall on 3 additional days. Of the participants who agreed, 26 were assigned to group 1 and 24 to group 2, following a counterbalanced crossover design. Hence, participants in group 1 completed three 24-hour recalls exactly a week before the APPetite-food record was used. In group 2, participants completed three 24-hour recalls exactly a week after the APPetite-food record was used. The same weekdays, the week before or after, were assessed. Both groups received the same training to familiarize themselves with the 24-hour recall and the APPetite-food record. Participants received €30 (US $36.52) to participate in the validation study.

#### Statistical Methods

Habitual energy and macronutrient intake assessed through the APPetite-food record was compared with habitual dietary intake assessed through the 24-hour recall.

Habitual energy and macronutrient intake from the two methods were compared at the group level using two-tailed paired *t* tests (for normally distributed data including energy and carbohydrates) and Wilcoxon signed-rank tests (for skewed data including protein, fat, sugar, and fiber). Agreement between the two methods at the individual level was assessed using Bland-Altman analysis of the mean differences [57]. For this, the difference between the two methods (y-axis) is plotted against the mean of the two methods (x-axis) for each participant. For reference, the mean difference between the two methods across all participants and the limits of agreement (LoA) estimated by the mean difference above and below 1.96 SD of the differences are shown in the plot. Thus, a systematic bias throughout the range of measurements can be identified. Acceptable LoA must be predefined. We predefined acceptable LoA for energy and macronutrient intake as 10% of the group mean across the two methods. Daily energy intake from the APPetite-food record (not normally distributed on days 2 and 3 of the EMA assessment) was compared with TEE (normally distributed) through a two-tailed paired *t* test for the first day and Wilcoxon signed-rank tests for the second and third days. Paired *t* tests (two-tailed) were calculated to compare mean TEE and habitual energy intake from the APPetite-food record and the 24-hour recall. The α level was set to .05. The analyses were performed using R 3.6.1 with RStudio.

## Results

### Feasibility

A total of 98.28% (3703/3768) of all scheduled prompts were delivered. The failure of prompt delivery was either due to technical problems or because the smartphone was switched off. Overall, 80.31% (3026/3768) of the prompts were answered completely. In total, 0.9% (34/3768) of prompts were registered as incomplete as a result of technical problems or extensive breaks during prompt completion. A total of 1.81% (68/3768) of prompts were dismissed, and 15.26% (575/3768) of prompts were ignored. The relatively large proportion of ignored prompts was, to some extent, a result of participants unintentionally leaving their smartphone at home or in another room. Furthermore, a number of participants reported that they had missed the first prompt or prompts of the day, as they were still sleeping.

Overall mean compliance (percentage of complete prompts within received prompts) was 81.73% (SD 21.65%). The compliance rate of 67.5% (106/157) of participants was above 80% (LORA: 94/136, 69.1%; PROUD: 4/7, 57%; and BipoLife-A1: 8/14, 57%). The mean compliance rate was 81.56% (SD 25.98%) on the first day, 83.28% (SD 23.55%) on the second day, and 79.97% (SD 25.8%) on the third day. The Friedman test showed no significant difference in compliance among the 3 days (*X*^2^_2_=3.6; *P*=.17), indicating no decline in motivation.

Compliance was highest in the LORA cohort and lowest in the PROUD cohort (see cohort means and SDs in Table 2). However, the Kruskal-Wallis rank sum test showed that compliance of the 3 cohorts did not differ significantly (*X*^2^_2_=0.7; *P*=.72).

Female participants had, on average, a compliance of 83.95% (SD 19.02%). The mean compliance for male participants was 77.83% (SD 25.34%). The Wilcoxon rank sum test found no gender difference in compliance (*P*=.16). No significant correlation was found between age and compliance (ρ=0.13; *P*=.12).

Participants responded to prompts after a mean of 189.32 seconds (SD 388.65). Responding to 70.54% (2157/3058) of all prompts was started within the first 60 seconds after the first prompt signal. The mean time needed to complete a single prompt was 122.63 seconds (SD 70.01). The prompt response latency and response duration for each of the 3 cohorts are shown in Table 3.

**Table 2:** Mean number and percentage of complete, incomplete, dismissed, and ignored prompts within received prompts for the total sample and each cohort.

Samples			Complete		Incomplete		Dismissed		Ignored
**Total (N=157), mean (SD)**									
	Values	19.27 (5.32)		0.22 (0.44)		0.43 (1.07)		3.66 (4.79)	
	Percentage	81.73 (21.65)		0.93 (1.89)		1.82 (4.48)		15.52 (20.08)	
**LORA^a^** **(n=136), mean (SD)**									
	Values	19.43 (5.04)		0.23 (0.46)		0.4 (1.05)		3.5 (4.46)	
	Percentage	82.48 (20.41)		0.98 (1.95)		1.71 (4.4)		14.83 (18.7)	
**PROUD^b^** **(n=7), mean (SD)**									
	Values	17.14 (7.73)		0.43 (0.54)		0.86 (1.46)		5.57 (6.95)	
	Percentage	71.43 (32.22)		1.79 (2.23)		3.57 (6.09)		23.21 (28.95)	
**BipoLife-A1 (n=14), mean (SD)**									
	Values	18.86 (6.76)		0 (0)		0.5 (1.09)		4.29 (6.63)	
	Percentage	79.59 (27.46)		0 (0)		2.08 (4.55)		18.33 (27.8)	

^a^LORA: Longitudinal Resilience Assessment.

^b^PROUD: Prevention of Comorbid Depression and Obesity in Attention-Deficit/Hyperactivity Disorder.

**Table 3:** Response latency and duration for a single prompt, reporting latency of the food record, and recording duration for the food record per day for the total sample and each cohort.

Variables		Total	LORA^a^	PROUD^b^	BipoLife-A1
**Prompts (s), mean (SD)**					
	Latency	189.32 (388.65)	179.38 (375.82)	242.24 (447.85)	265.24 (469.8)
	Duration	122.63 (70.01)	119.13 (64.21)	175.12 (144.31)	134.01 (64.07)
**Food record (min), mean (SD)**					
	Latency	58.35 (127.52)	50.82 (115.8)	147.02 (197.85)	116.99 (190.16)
	Duration	17.66 (8.66)	17.8 (8.57)	22.17 (10.86)	14.26 (7.56)

^a^LORA: Longitudinal Resilience Assessment.

^b^PROUD: Prevention of Comorbid Depression and Obesity in Attention-Deficit/Hyperactivity Disorder.

Dietary data of 8.8% (12/136) LORA, 14% (1/7) PROUD, and 14% (2/14) BipoLife-A1 participants had to be excluded, as the number of recorded meals or entered foods was evidently too low or entries were incomplete or implausible. In addition, 3 LORA participants had no food entry on 1 day. However, the remaining 2 days were recorded sufficiently well to be included. Included participants (n=142) recorded a total of 2969 eating and drinking events. In total, 3.03% (90/2969) entries were registered as incomplete, mainly due to technical problems. Participants entered on average 7.02 (SD 3.33) eating and drinking events per day (first day: mean 7.49, SD 3.14; range 2-17; no data available for 1 participant; second day: mean 7.08, SD 3.43; range 2-22; third day: mean 6.49, SD 3.37; range 2-17; no data available for 2 participants).

The mean latency from food intake to food recording was 58.35 (SD 127.52) minutes. Latency increased over the course of the 3 days (first day: mean 53.51, SD 72.01 min; second day: mean 69.5, SD 88.1 min; third day: 90.81, SD 116.12 min). The mean time to complete the food record of one day was 17.66 (SD 8.66) minutes. On the first day, participants took 21.01 (SD 9.68) minutes, on the second day they took 17.22 (SD 7.76) minutes, and on the third day they took 14.67 (SD 7.16) minutes. The cohort-specific food record latencies and recording durations are presented in Table 3.

The accelerometer records of 2 participants stopped during the second day. It is unknown if this was due to technical problems or because participants connected the sensor to a computer that instantly stopped the recording. In total, 11 participants did not wear the sensor on at least one day or stopped wearing it before the end of the 7-day assessment period. On average, participants (N=157 including the abovementioned) wore the sensor for 6 days 3 hours and 57 minutes (mean 8876.96, SD 1815.36 min; range 771-10,403). Hence, the mean compliance was 88.07% (SD 18.01%).

### Usability

The SUS total score was 61.9 (SD 17.79; range 17.5-97.5) out of 100. The SUS score of the LORA cohort (n=67) was 61.23 (SD 16.8; range 17.5-95). The lowest usability with an SUS score of 60 (SD 24.08; range 32.5-92.5) was rated by the PROUD cohort (n=6). The highest SUS score was found for the Bipolife-A1 cohort (n=11), with a score of 67.05 (SD 20.97; range 22.5-97.5). However, the 3 cohorts did not differ in the ratings of usability according to a one-way analysis of variance (*F*_2,81_=0.54; *P*=.59).

Female participants (mean 62.82, SD 17.36) scored usability on average marginally higher than male participants (mean 60.17, SD 18.77; t_82_=0.65 [two-tailed]; *P*=.52). Age and usability were not significantly negatively correlated (ρ=−0.18; *P*=.10). Compliance and rated usability showed no significant correlation (ρ=0.13; *P*=.26).

### Validity

Habitual intake of energy, protein, fat, carbohydrates, sugar, and fiber assessed through the APPetite-food record and the 24-hour recall are shown in Table 4. All nutritional intake was higher for the APPetite-food record. The difference between the two methods is significant for energy, protein, fat, and fiber intake (Table 4).

With regard to possible order effects, both groups (APPetite-food record first and 24-hour recall first) showed higher energy intake assessed through the APPetite-food record (group 1: 8494 kJ/2029 kcal; group 2: 9327 kJ/2228 kcal) compared with the 24-hour recall (group 1: 7881.23 kJ/1882 kcal; group 2: 8086.01 kJ/1931 kcal).

**Figure 3:**
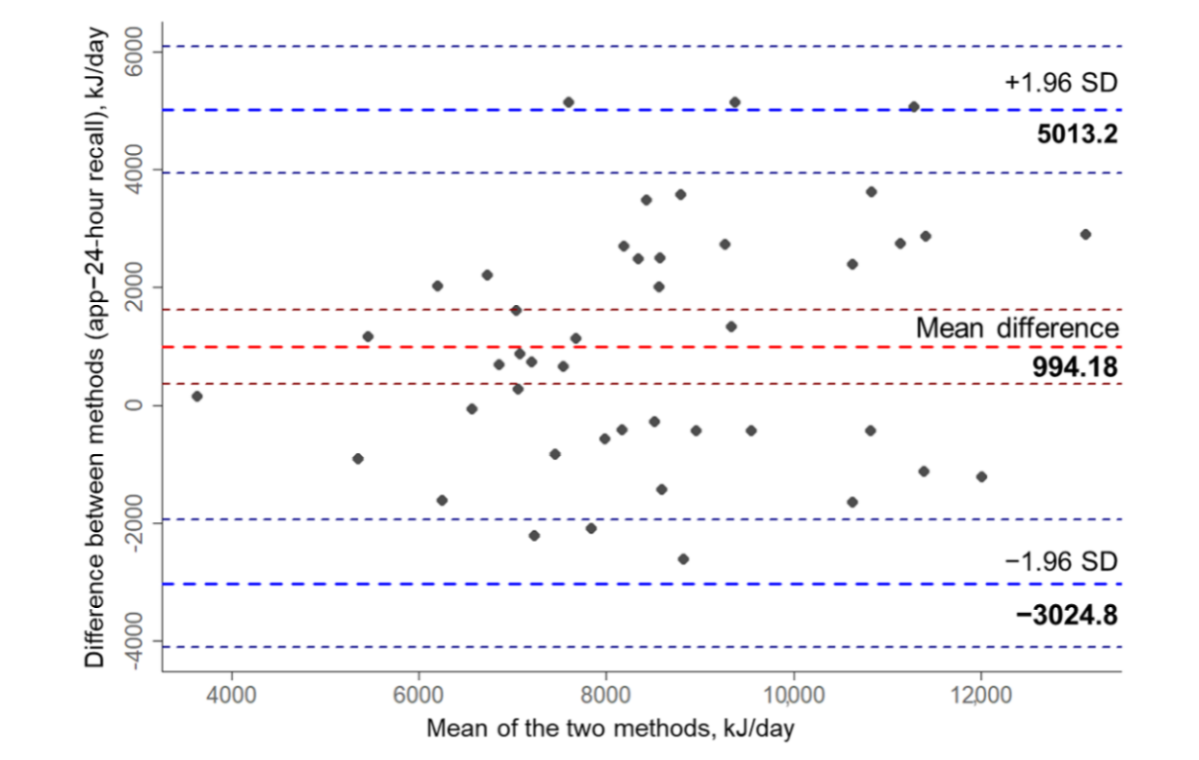
Bland-Altman plot assessing agreement between habitual energy intake in kJ per day captured by the APPetite-food record and the 24-hour recall (red line: mean difference=app–24-hour recall; dark red lines: 95% CI of mean difference; blue lines: lower and upper limits of agreement; dark blue lines: 95% CI of lower and upper limits of agreement).

Agreement between the two methods at the individual level was investigated through Bland-Altman plots for energy and macronutrient intake. Mean energy difference between the APPetite-food record and the 24-hour recall was 994.18 kJ (95% CI 370.8-1617.6). A normal distribution of the difference was observed. Figure 3 shows the Bland-Altman plot of the habitual energy intake. The LoA are −3024.841 (95% CI −4104.6 to −1945.1) to 5013.2 (95% CI 3933.4-6093) and therefore larger than the predefined acceptable LoA of 849 kJ.

Bland-Altman analyses for protein, fat, carbohydrate, sugar, and fiber intake can be found in Multimedia Appendix 4 [57]. All LoA exceeded the predefined acceptable LoA.

**Table 4:** Mean habitual intake of energy and macronutrients from the APPetite-food record and the 24-hour recall; mean difference between the two methods; paired *t* tests (two-tailed) for normally distributed data including energy and carbohydrates; and Wilcoxon signed-rank tests for skewed data, including protein, fat, sugar, and fiber.

Dietary intake per day	APPetite-food record, mean (SD)	24-hour recall, mean (SD)	Mean difference (SD)	*t* test (*df*)	*P* value
Energy (kJ/day)	8987.11 (2404.65)	7992.93 (2002.61)	994.18 (2050.52)	3.21 (43)	.003^a^
Energy (kcal/day)	2146.42 (574.5)	1909.16 (478.8)	237.26 (489.94)	3.21 (43)	.003
Protein (g/day)	80.77 (27.6)	69.68 (24.72)	11.09 (22.63)	N/A^b^	.004^a^
Total fat (g/day)	92.25 (32.54)	76.95 (26.54)	15.3 (29.41)	N/A	.002^a^
Carbohydrate (g/day)	228.81 (63.12)	212.99 (52.65)	15.82 (64.03)	1.64 (43)	.11
Sugars (g/day)	81.21 (32.6)	76.97 (29.76)	4.24 (31.42)	N/A	.40
Fiber (g/day)	25.86 (9.22)	23.3 (8.5)	2.56 (7.43)	N/A	.04^c^

^a^*P*<.01.

^b^N/A: not applicable to Wilcoxon signed-rank test.

^c^*P*<.05.

Energy intake from the APPetite-food record was significantly lower than the TEE estimated from accelerometry on the first day (t_43_=5.33; *P*<.001; TEE mean 2425.4, SD 468.2; app mean 1897.53, SD 616.32), but did not significantly differ on days 2 and 3: day 2 (*P*=.051; TEE mean 2442.04, SD 447.5; app mean 2242.94, SD 769.78) and day 3 (*P*=.23; TEE mean 2435.6, SD 482.9; app mean 2317.77, SD 780.6). Mean TEE estimated from 7 days of accelerometry was 2417.8 kcal (SD 410) compared with the habitual energy intake of 2146.42 kcal (SD 574.5) from the APPetite-food record and 1909.16 kcal (SD 478.8) from the 24-hour recall. Paired *t* tests (two-tailed) showed that habitual energy intake was underestimated by both methods when compared with TEE: APPetite-food record (t_43_=3.40; *P*=.002) and 24-hour recall (t_43_=6.33; *P*<.001).

## Discussion

### Principal Findings

The APPetite-mobile-app was developed to capture complex dietary intake, objectively recorded PA, and related factors for studying microtemporal, within-person processes underlying eating behavior and PA in daily life. This study evaluated the feasibility and usability of the EMA tool as well as the validity of the APPetite-food record. The APPetite-mobile-app demonstrated good feasibility. Compliance with responding to prompts and wearing the accelerometer was above 80%, and reasonable response times and latencies were found for the prompts as well as the food record. Usability was rated moderate, with a mean SUS score of 61.9. Large differences in energy and macronutrient intake assessed with the APPetite-food record versus the 24-hour recall were found at the group and individual levels, whereby the APPetite-food record captured higher dietary intakes. Energy intake was assessed fairly accurately by the APPetite-food record on the group level on 2 of 3 days when compared with TEE. The comparison of habitual energy intake to mean TEE showed that the 24-hour recall underestimated energy intake to a larger degree than the APPetite-food record. These results indicate that the discrepancies between the two dietary assessment methods do not imply a lack of validity of the APPetite-food record; rather, they indicate a more accurate dietary assessment compared with the 24-hour recall and therefore provide the first evidence that the APPetite-food record is a valid tool for capturing complex dietary intake.

### Comparison With Previous Work

The good feasibility of EMA tools is crucial to ensure unbiased data collection and prevent systematic missing data. Compliance rates are an important indicator of feasibility. Although there is no official criterion indicating good compliance, Stone and Shiffman [58] proposed compliance rates above 80% to be acceptable. However, they emphasized the arbitrariness of this criterion and the need to define acceptable compliance ranges for each study individually, especially when noncompliance may be systematic and not random. The mean prompt compliance in our study was above 80% and can therefore generally be rated as good. These good compliance rates may be partly due to the notifications participants received when falling below the 80% threshold and the incentive to be included in raffles when reaching a compliance rate of 80% or above.

Furthermore, the results demonstrate that prompt compliance did not decrease over the course of 3 days. In other EMA studies that assessed more than 3 days, response rates declined substantially (eg, 40% from 63% on day 1 to 23% on day 7), even with only 4 prompts per day [36]. As studying microtemporal processes requires an illustration of a day in high resolution, it is more important to focus on a larger number of completed prompts per day compared with a large number of EMA assessment days. On the basis of our constant compliance rate over 3 days, the length of the EMA assessment seems feasible, and no decline in motivation was evident.

We found marginally lower prompt compliance rates in the clinical cohorts than in the healthy cohort. In a study by Porras-Segovia et al [59] comparing EMA compliance rates from suicidal patients and student controls, lower compliance was found for the clinical sample. These findings were consistent with our results. However, Porras-Segovia et al [59] found a significant difference between patients and healthy controls, which was not the case in our study. These results suggest that the prompt schedule of the APPetite-mobile-app is equally well suited for healthy individuals and patients with a mental disorder.

A mean of 2.04 (SD 1.17) minutes was needed to complete 1 prompt. In accordance with the high prompt compliance rate, the response duration of the prompts can be considered feasible. Responding to a prompt was initiated on average 3.16 (SD 6.48) minutes after the first prompt signal. Short prompt latencies are essential to guarantee the momentary nature of the response and should therefore be taken into account thoroughly. However, most studies have not reported prompt latencies [60,61]. Some studies have predefined response windows. This ensures the momentary nature of the response but can cause lower compliance rates, for example, 69% in a study with an 8-minute response window [62]. We chose to allow a longer response period and prompt postponement of up to 25 minutes to reduce participants’ burden and maintain high compliance. Nevertheless, participants were instructed to respond to EMA prompts instantly, if possible. Considering that we allowed responses up to 30 minutes after the first prompt signal, the mean latency of just over 3 minutes is short and underlines the feasibility of the prompts.

Compliance with the food record cannot be directly determined, as it is not possible to differentiate between someone not recording a food item because of noncompliance or because of not actually consuming it. However, other quality measures could also be used. The time spent reporting daily dietary intake or the number of recorded eating and drinking occasions per day can be used for quality checks. On average, participants needed 17.6 minutes to complete the food record of 1 day. Other technology-based tools for assessing dietary intake show similar times to complete, ranging between 13 and 45 minutes [20]. Participants entered on average 7 eating and drinking events per day. This number is in line with a previous study that found a mean of 20.7 eating and drinking occasions per individual over a 3-day period [29].

The APPetite-food record was developed to record food intake in real time or near real time. Therefore, it is important to consider the amount of time between food intake and recording. Foods and drinks were recorded on average 58.35 minutes after intake. This shows that participants did not wait until the evening to record all eating and drinking occasions for 1 day. Hence, food intake was recorded in real time or near real time.

The food recording behavior of our participants suggests that the APPetite-food record is feasible. However, we noticed that the participants’ motivation was crucial for successfully capturing sufficient and accurate dietary data. Training is needed to ensure that participants understand the importance of food recording in real time or near real time. Furthermore, participants reported that receiving detailed dietary feedback at the end of the study increased their motivation to enter food intake accurately.

As expected from previous studies [63], a high compliance rate (88.07%) for the accelerometer worn on the wrist were found. All measures of feasibility regarding prompts, the APPetite-food record, and the accelerometer indicate that the APPetite-mobile-app is a feasible EMA tool.

In addition to good feasibility, usability is an important criterion that should be considered when developing new EMA tools. The usability of the APPetite-mobile-app was rated as moderate, with an SUS score of 61.9 out of 100. In a previous study, the usability of the top 7 iPhone operating system and Android diet-tracking apps was assessed [22]. The usability of 2 apps was rated even lower than the APPetite-mobile-app (Lose It!=59.2; MyDietCoach=46.7). However, a comparison is difficult as these tools focus purely on dietary assessment. The SUS score of the APPetite-mobile-app was rated on the basis of both dietary assessment and EMA prompts. The relatively low usability of our tool can be explained by the fact that it is a scientific device and was therefore developed independently without professional app developers. High costs are involved in the professional development of an app. For this reason, we chose the platform movisensXS to independently develop the app. Although movisensXS has many configuration options, it still has its limitations. For example, a search function within the food record cannot be implemented. The app was developed for scientific purposes only and not for consumer use. However, usability challenges have been reported even for commercial tools; for instance, only 20% of participants would continue to use MyFitnessPal after study participation [64]. Even though the usability of the APPetite-mobile-app was rated relatively low, no negative effect on feasibility, including compliance, was evident. Therefore, an improvement in usability is desirable but not essential for its use in scientific research.

A food record was incorporated into the APPetite-mobile-app to capture complex dietary intake in real time or near real time. An evaluation of validity was needed to test whether the APPetite-food record accurately assessed dietary intake. Hence, the APPetite-food record was compared with a 24-hour recall and TEE estimated from nondominant wrist accelerometry.

With regard to relative validity, low agreement between habitual dietary intake measured by the APPetite-food record and the 24-hour recall was found at both the group and individual levels. At the group level, energy, protein, fat, and fiber intake from the APPetite-food record was significantly higher than the 24-hour recall. Wide LoA, which exceeded the predefined acceptable LoA, were found for energy, protein, fat, carbohydrate, sugar, and fiber intake at the individual level. One could argue that these discrepancies indicate a lack of validity in the APPetite-food record. However, even though 24-hour recalls are frequently used as the established reference method when assessing relative validity, the true intake remains unknown [65]. Therefore, possible reasons for this discrepancy must be taken into account for both methods. Most validation studies that compared an EMA dietary assessment tool with a 24-hour recall found lower values for energy intake as well as intake of some macronutrients assessed through the EMA tool on the descriptive or even statistical level (eg, [64] for energy [statistical], proteins [statistical], fat [statistical], carbohydrates [statistical], and sugar [statistical]; [65] for energy, protein, fat, and carbohydrates; [66] for energy and fat [statistical], not for proteins and carbohydrates; [67] for energy, fat, and carbohydrates, not for proteins; [68] for energy, protein, sugar, and fat, not for carbohydrates and fiber, no statistical hypothesis test reported; [69] for energy, fat [statistical], carbohydrates, and fiber, not for proteins). This was not the case in our study. Habitual energy, protein, fat, carbohydrate, sugar, and fiber intake was higher when assessed with the APPetite-food record on the descriptive or even statistical level, indicating a lower degree of underreporting. This leads to the conclusion that the APPetite-food record could be a more precise dietary assessment method than the 24-hour recall.

This interpretation is underlined by the comparison of energy intake and TEE estimated from accelerometry. Energy intake from the APPetite-food record was not significantly different from TEE on 2 of 3 days, indicating that the APPetite-food record assesses energy intake fairly accurately at the group level. However, the comparison with the mean TEE showed that both methods underestimated habitual energy intake. In this context, it must be mentioned that over one-third of the participants in the validation study (17/44, 39%) indicated that they are currently trying to lose weight. Therefore, the discrepancy between the TEE and the reported energy intake could, to some extent, be due to diet and weight loss. However, the 24-hour recall underestimated habitual energy intake to a greater extent. Inaccurate estimates of energy intake captured by 24-hour recalls have been reported in previous studies [70]. A reason for the improved reporting accuracy in dietary assessments in real time or near real time compared with retrospective assessments could be the minimized retention interval [71,72]. Memory effects can cause bias in retrospective dietary assessments, as the demand for memory increases simultaneously with the retention interval. Memory lapses can cause two types of errors in the context of 24-hour dietary recalls: the failure to recall foods actually consumed (errors of omission) and the reporting of foods that were actually not consumed during the recalled day (errors of intrusion) [73]. Furthermore, incorrect estimations of portion sizes have been reported to constitute the largest measurement error in 24-hour recalls [73]. This error is closely related to memory bias, as consumed amounts must not only be accurately estimated but also be correctly remembered [74]. Food records in real time or near real time can minimize memory errors [74]. In a recent study, 65% of participants reported that remembering meal items and portion sizes was easier in a progressive assessment than in a traditional retrospective 24-hour recall [75]. Nonetheless, food records in real time or near real time are also affected by potential bias, which was also shown in our study as underestimation of food intake became evident. In particular, the change in dietary intake as a result of recording it has to be taken into account. Participants may choose not to eat complex meals or eat less to avoid extensive and time-consuming records [74]. Furthermore, keeping a record of food intake in daily life can be burdensome. Participants may not be able to record everything eaten due to other commitments. However, our results suggest that the impact of reactivity and high burden on the APPetite-food record might be smaller than the effect of memory loss on 24-hour recalls.

The results of the evaluation of validity indicate that the APPetite-food record might assess dietary intake more accurately than the 24-hour recall and capture daily energy intake fairly accurately at the group level. Nevertheless, both dietary assessment methods seem to underestimate habitual energy intake.

### Strengths and Limitations

A common validation approach is the assessment of relative validity, which compares a novel tool to an established dietary assessment method. However, most of the available validation studies show methodological issues as they assess relative validity on overlapping days [64-68] and do not use a counterbalanced crossover design [69]. Assessing overlapping days can lead to an overestimation of agreement between two self-report methods, as recording dietary intake actively throughout the day may improve memory for completing the 24-hour dietary recall of the same day. Empirical evidence for the overestimated agreement has been found, detecting improved accuracy of 24-hour recalls of days when diet was tracked throughout [65]. A further problem becomes apparent in studies that do not assess overlapping days. When two methods are used one after another, order effects can bias the assessment. However, most studies did not control for possible order effects [69]. We were able to counteract these methodological issues by choosing a counterbalanced crossover study design that assessed no overlapping days. A counterbalanced crossover design is crucial for controlling learning, boredom, and other unwanted order effects. We understand this to be the most significant strength of our validation study.

One limitation of our validation study is due to the fact that dietary intake varies from day to day. Bland and Altman call this case “method where true value varies” [76]. When the true value varies, measurements of two methods have to be taken at the same time point to obtain an accurate estimate of agreement [77]. In the context of dietary assessment methods that would translate to assessing food intake using two methods on the same day. However, because an inflated agreement when assessing overlapping days is likely to occur [26], as mentioned earlier, this does not represent a suitable approach. Therefore, we were not able to compare dietary intake on a day level (eg, Thursday compared with Thursday the week before or after) and chose to compare habitual dietary intake instead. However, when comparing habitual dietary intake, two aspects must be considered: (1) the target of interest of the APPetite-mobile-app is not regular or habitual food intake but rather microtemporal dynamics of food intake in daily life. Using habitual intake as the measure of comparison sets aside this fact and might therefore not be the most appropriate measure for the evaluation of validity. (2) Day-to-day variability in dietary intake represents a problem when assessing habitual intake. It could be argued that capturing 3 days to operationalize habitual intake is not sufficient to obtain an accurate estimate.

Many studies that use Bland-Altman agreement analyses to evaluate the validity of food records in real time or near real time have inaccuracies. To the best of our knowledge, our study is the only one that has a predefined acceptable LoA. These pre-established limits are necessary to avoid misleading interpretations. A consensus on the acceptable LoA for dietary intake is desirable. This will improve the comparability of the results from studies assessing relative validity. Furthermore, the use of established but biased dietary assessment methods, such as 24-hour recalls, to study relative validity should be questioned critically. New approaches to evaluate the validity of food records in real time or near real time are needed.

Our findings are limited because of the lack of control for possible weight changes during study participation. The comparison of TEE and energy intake is based on the assumption that energy expenditure is equal to energy intake. However, this assumption is valid only for weight-stable individuals.

Two further limitations concern the APPetite-food record itself: (1) nutritional values are generated manually, which is time-consuming and can be error-prone. Automated generation is preferable. (2) The APPetite-food record relies on self-reports of dietary intake. Self-reports are subjective and therefore more likely to be biased. To add a more objective component to the dietary assessment, photos of the foods and drinks consumed could be taken in addition to self-reports.

The strength of our assessment of feasibility and usability is that the sample of healthy participants was enriched with data from patients suffering from a mental disorder. Therefore, it was possible to show that the APPetite-mobile-app is equally feasible and usable in this population. This finding is particularly important as diet and PA play an important role in mental health. This opens up the possibility of studying microtemporal, within-person processes of diet, PA, and related factors in psychiatric patients, which is crucial for the understanding of the link among diet, PA, and mental health. However, the unequal sample sizes of the 3 cohorts limit the results. This is of concern in the context of cohort comparisons, as well as the interpretation of the means of the total sample. Furthermore, a selection bias could be present, as the participants were exclusively recruited from 3 existing study cohorts.

### Recommendations for Future Studies

The development of novel EMA tools for assessing microtemporal processes of diet, PA, and related factors is required. Studies comparing these new EMA tools are needed to establish empirical evidence on which assessment approaches are most effective in the study of microtemporal processes. Future EMA studies should consider that participants’ motivation is the key to success, especially when complex dietary intake is assessed. Therefore, participants’ burden needs to be kept minimal, and incentives for prompt responding and food recording, such as dietary feedback and raffle inclusions, are essential.

New technologies and wearable sensors are a promising advancement in the area of dietary assessment in naturalistic settings, as they can passively detect eating behavior [78]. They can be used for longer assessment periods because they require minimal user interaction. These sensors will improve the validity of self-reported dietary assessments to a great extent. We believe they will soon be of tremendous relevance, especially for the assessment of microtemporal processes of diet in daily life.

### Conclusions

The APPetite-mobile-app is a promising tool for studying microtemporal, within-person processes of diet, PA, and related factors in real time or near real time and is, to the best of our knowledge, the first of its kind. First evidence supports that the APPetite-mobile-app is feasible and the APPetite-food record is a valid tool for capturing complex dietary intake. We hope this motivates other researchers to use EMA to capture complex dietary intake, PA, and associated factors in daily life, and it initiates a discussion about feasible, usable, and valid methods to assess these dynamics. Assessment strategies need to be developed, shared, and discussed to advance the research field. A solid empirical foundation regarding within-person, microtemporal associations of diet, PA, and associated factors is needed for the development of personalized lifestyle modification interventions, such as intensively adaptive interventions or just-in-time adaptive interventions [10].

## Appendix

Multimedia Appendix 1 APPetite-food record and nutritional value generation.

Multimedia Appendix 2 Reasons for food intake.

Multimedia Appendix 3 Prompt measures and items.

Multimedia Appendix 4 Bland-Altman analysis of protein, fat, carbohydrate, sugar, and fiber intake.

## Abbreviations

EMA: ecological momentary assessment
LoA: limits of agreement
LORA: Longitudinal Resilience Assessment
PA: physical activity
PROUD: Prevention of Comorbid Depression and Obesity in Attention-Deficit/Hyperactivity Disorder
SUS: System Usability Scale
TEE: total energy expenditure
