# Supplementary material for: Studying Microtemporal, Within-Person Processes of Diet, Physical Activity, and Related Factors Using the APPetite-Mobile-App: Feasibility, Usability, and Validation Study
Source: J Med Internet Res. 2021 Jul 5;23(7):e25850. doi: 10.2196/25850 (PMC8406112; doi:10.2196/25850)
Supplement: Multimedia Appendix 1 [file jmir_v23i7e25850_app1.docx]

**Multimedia Appendix 1.** APPetite-food record and nutritional value generation.

Participants are asked to enter foods and drinks as soon as possible after consuming them. The home-screen of the APPetite-mobile-app displays 5 buttons “breakfast”, “lunch”, “dinner”, “snack”, and “drink”. Pressing the appropriate meal button triggers the initiation of the APPetite-food record. After confirming the intent to start entering a meal as all subsequent steps are mandatory, participants set the time of the meal on a digital 24-hour clock (hh:mm). Participants are then redirected to a list of 16 categories of which they select the applicable ones. The subcategories as well as the food and drink items of each selected category are displayed subsequently. If the button “drink” is pressed, participants can only choose from the drink category. After selecting all relevant food and drink items, each item will be presented again for the assessment of the amount consumed. Participants can specify the amount by entering the number of consumed standard portions (eg, 200 ml glass for water) or an exact amount in milliliters or grams. To ensure participants do not forget to enter certain foods or drinks, a reminder to record everything (eg, milk when having cereals) is presented. After the reminder participants indicate whether they (1) have entered everything, (2) want to add something else from the list, or (3) want to use the Not-on-the-list-option. Within the Not-on-the-list-option participants are asked to describe the food and amount consumed as accurately as possible. In the last step, participants select the predominant reason for the current food or drink intake out of 19 presented reasons. These reasons were adopted from previous studies [1–5], translated to German and adapted to incorporate reasons for drinking (see Multimedia Appendix 2).

At 9 PM, the APPetite-mobile-app initiates an end-of-the-day prompt asking if all consumed foods and drink of the day have been recorded. If participants deny this question, they are requested to add missing meals following the described 6-step process.

The APPetite-food record includes 14 food-related categories, 1 drink category, and a Not-on-the-list-category. The food-related categories are divided into 31 subcategories and into around 500 food items overall. The drink category differentiates a total of 40 drinks in 2 subcategories: nonalcoholic and alcoholic drinks. The food and drink items are predominantly generic. To facilitate the search of certain foods and drinks, some items are present in more than one category (eg, milk in dairy products and nonalcoholic drinks).


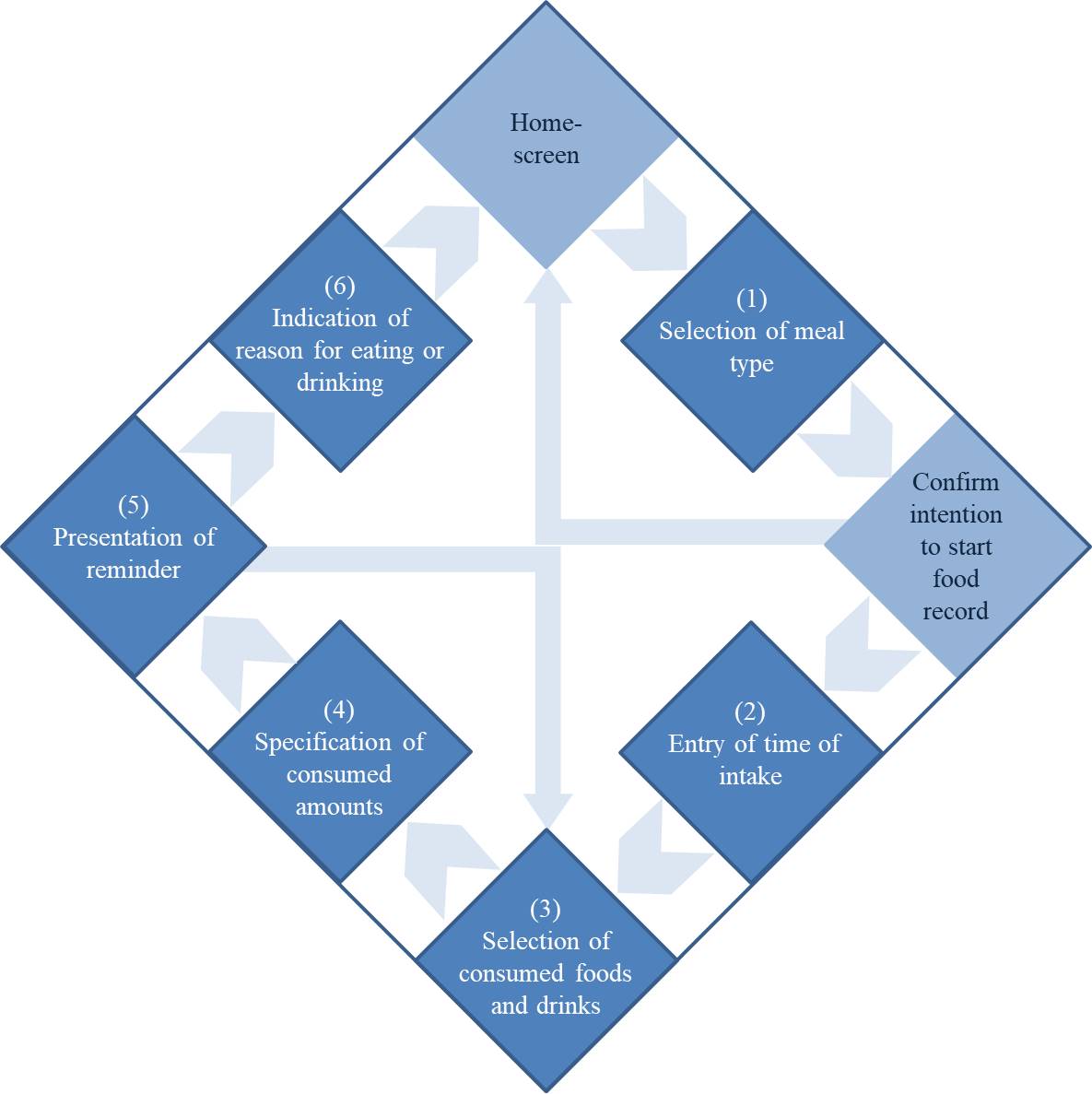


**Figure 1.** Illustration of the six-step process of the APPetite-food record.

A few additional features were implemented to improve the accuracy of the APPetite-food record. These features address further details about entered foods or the consumption of commonly added foods: (1) Preparation method of fruits and vegetables (eg, raw, cooked, or fried), (2) Caffeine content of coffee and tea beverages, (3) Type of fruit of juices, nectars or spritzers, (4) Added sugar, honey, sweetener pills or sweetener liquid to hot drinks, and (5) Added seeds (eg, sunflower seeds) to salads.

Participants cannot modify food records retrospectively. As compliance of the food record cannot be assessed directly, we provide dietary feedback to our participants to increase motivation to enter all foods and drinks completely and truly.

##### Nutritional value generation

The APPetite-food record captures complex dietary information. However, it does not allow the automated generation of nutritional values. Therefore, we created a workflow starting with the data download and data preprocessing, followed by the data plausibility check and the data transfer, resulting in the generation of nutritional values. Data preprocessing is done using RStudio extracting all consumed food and drink items and their amounts from the movisensXS output file. The extracted dietary data are then checked for plausibility. Questionable entries are identified (eg, “100 apples”) and reviewed with the participant in the second in-person session. Furthermore, additional information (eg, product brand) on recorded generic foods is acquired if possible. The checked and corrected dietary data are then transferred to myfood24-Germany, a 24-hour dietary recall [6], by trained staff. Data plausibility check, data correction, and data transfer are done according to the 4-eyes principle to minimize data loss and errors.

1. Cleobury L, Tapper K. Reasons for eating “unhealthy” snacks in overweight and obese males and females. J Hum Nutr Diet. 2014;27(4):333–341. PMID: 24134077

2. Verhoeven AAC, Adriaanse MA, de Vet E, Fennis BM, de Ridder DTD. It’s my party and I eat if I want to. Reasons for unhealthy snacking. Appetite. 2015;84:20–27. PMID: 25261101

3. Schüz B, Papadakis T, Ferguson SG. Situation-specific social norms as mediators of social influence on snacking. Heal Psychol. 2018;37(2):153–159. PMID: 29154607

4. Renner B, Sproesser G, Strohbach S, Schupp HT. Why we eat what we eat. The Eating Motivation Survey (TEMS). Appetite. 2012;59(1):117–128. PMID: 22521515

5. Evers C, Adriaanse M, de Ridder DTD, de Witt Huberts JC. Good mood food. Positive emotion as a neglected trigger for food intake. Appetite. 2013;68:1–7. PMID: 23602962

6. Koch SAJ, Conrad J, Hierath L, Hancock N, Beer S, Cade JE, et al. Adaptation and evaluation of myfood24-germany: A web-based self-administered 24-h dietary recall for the german adult population. Nutrients. 2020;12(1):1–15. PMID: 31935885
