# Supplementary material for: Studying Microtemporal, Within-Person Processes of Diet, Physical Activity, and Related Factors Using the APPetite-Mobile-App: Feasibility, Usability, and Validation Study
Source: J Med Internet Res. 2021 Jul 5;23(7):e25850. doi: 10.2196/25850 (PMC8406112; doi:10.2196/25850)
Supplement: Multimedia Appendix 2 [file jmir_v23i7e25850_app2.docx]

**Multimedia Appendix 2.** Reasons for food intake.

Bitte wählen Sie die zutreffendste Aussage aus:

Ich habe gegessen/getrunken, …

… weil ich hungrig/durstig war. [1]

… um später nicht hungrig/durstig zu sein. [1]

… weil ich Energie gebraucht habe. [1]

… weil ich normalerweise zu dieser Zeit esse/trinke. [1]

… weil das Essen/Getränk so verlockend aussah/gerochen hat. [1]

… weil ich traurig war. [1]

… weil mir langweilig war. [1]

… weil ich gestresst war. [1]

… weil ich mich einsam gefühlt habe. [2]

… weil ich glücklich war. [3]

… um jemand anderem/anderen Gesellschaft zu leisten. [1]

… weil ich mich verpflichtet gefühlt habe, zu essen/trinken. [1]

… weil ich nicht aufhören konnte, an Essen/Trinken zu denken. [1]

… weil ich verhindern wollte, dass Essen/Getränke verschwendet/weggeschmissen wird/werden. [1]

… um einen besonderen Anlass zu feiern (Geburtstag, Traditionelles Fest, etc.) [4]

… weil ich Fernsehen/einen Film geschaut habe. [4]

… um mich selbst zu belohnen. [4]

… weil andere gegessen/getrunken haben. [5]

Ich kann mich nicht erinnern, mich entschieden zu haben, zu essen/trinken. – Ich habe einfach gegessen/getrunken. [1]

**Please select the most applicable statement:**

**I ate/drank …**

… because I was feeling hungry/thirsty. [1]

… to avoid being hungry/thirsty later. [1]

… because I felt I needed the energy. [1]

… because I usually eat/drink at this time. [1]

… because the food/drink looked/smelt so tempting. [1]

… because I was feeling fed up. [1]

… because I was feeling bored. [1]

… because I was stressed. [1]

… because I felt lonely. [2]

… because I was happy. [3]

… to keep somebody else/other people company. [1]

… because I felt obliged to. [1]

… because I couldn’t stop thinking about food. [1]

… because I wanted to avoid food/drinks going to waste. [1]

… to celebrate a special occasion (birthday, traditional celebration, etc.). [4]

… because I was watching television/a movie. [4]

… to reward myself. [4]

… because others ate/drank. [5]

I don’t recall deciding to eat/drink – I just found myself eating/drinking. [1]

1. Cleobury L, Tapper K. Reasons for eating “unhealthy” snacks in overweight and obese males and females. J Hum Nutr Diet. 2014;27(4):333–341. PMID: 24134077

2. Renner B, Sproesser G, Strohbach S, Schupp HT. Why we eat what we eat. The Eating Motivation Survey (TEMS). Appetite. 2012;59(1):117–128. PMID: 22521515

3. Evers C, Adriaanse M, de Ridder DTD, de Witt Huberts JC. Good mood food. Positive emotion as a neglected trigger for food intake. Appetite. 2013;68:1–7. PMID: 23602962

4. Verhoeven AAC, Adriaanse MA, de Vet E, Fennis BM, de Ridder DTD. It’s my party and I eat if I want to. Reasons for unhealthy snacking. Appetite. 2015;84:20–27. PMID: 25261101

5. Schüz B, Papadakis T, Ferguson SG. Situation-specific social norms as mediators of social influence on snacking. Heal Psychol. 2018;37(2):153–159. PMID: 29154607
