# Supplementary material for: Studying Microtemporal, Within-Person Processes of Diet, Physical Activity, and Related Factors Using the APPetite-Mobile-App: Feasibility, Usability, and Validation Study
Source: J Med Internet Res. 2021 Jul 5;23(7):e25850. doi: 10.2196/25850 (PMC8406112; doi:10.2196/25850)
Supplement: Multimedia Appendix 3 [file jmir_v23i7e25850_app3.docx]

**Multimedia Appendix 3.** Prompt measures and items.

The German items of the prompts which are used within the APPetite-mobile-app are shown in black. The corresponding English items are shown in grey. All changes that have been made to the original items of the cited publications are described and explained in italic. Psychometric properties (McDonald’s Omega for between- and within-subject reliability) are reported for all constructs/scales assessed with at least 3 items. The item order is not randomized within the prompts.

**Sleep**

The first prompt of each day assesses quantity and quality of the previous night’s sleep. The time of falling asleep the night before and the time waking up this morning is captured on a digital 24-hour clock (hh:mm). Additionally, participants rate the sleep quality on a 5-point scale from bad to good.

Wann sind Sie gestern eingeschlafen?

hh:mm

What time did you fall asleep yesterday night?

hh:mm

Wie gut haben Sie geschlafen?

| Schlecht | Eher schlecht | Teils-teils | Eher gut | Gut |
| --- | --- | --- | --- | --- |

How was your sleep?

| Bad | Quite bad | So-so | Quite good | Good |
| --- | --- | --- | --- | --- |

Wann sind Sie heute Morgen aufgewacht?

hh:mm

What time have you woken up today?

hh:mm

**Stress expectancy**

The intensity of expected stress of the present day is rated in the first prompt of each day on a visual analogue scale from 0 (not stressful at all) to 100 (very stressful).

Wie stressig wird der heutige Tag insgesamt Ihrer Erwartung nach werden?

0 - gar nicht stressig 100 - sehr stressig

How stressful do you expect today to be?

0 – not stressful at all 100 – very stressful

**Context**

Each prompt captures the context (main activity, social and physical context) a person was in right before the prompt. The assessment was adapted from Dunton, Liao, Intille, Huh, and Leventhal [1].

*„In a conversation” was added to the list of main activities as well as “laying” to the last question of the main activity assessment.*

Was haben Sie gemacht, bevor das Signal zur Abfrage ertönte? Wählen Sie Ihre Haupttätigkeit.

Lesen / Computer

Fernsehen / Film schauen

Essen / Trinken

Körperliche Aktivität / Sport

Gespräch

Andere Tätigkeit

What were you DOING right before the beep went off [Choose your main activity]?

Reading/Computer

Watching TV/movies

Eating/drinking

Physical activity/exercising

In a conversation

Other

If “Physical activity/exercising” was selected:

Welche Art von Körperlicher Aktivität/Sport haben Sie gemacht? Bitte tippen Sie Ihre Antwort ein.

___________________________________________________________

What type of PHYSCIAL ACTIVITY/EXERCISE? Please type in your answer.

___________________________________________________________

If “Other” was selected:

Was war Ihre Tätigkeit?

Telefonieren

Kochen / Hausarbeiten

Auto fahren

Kinderbetreuung / Kindern helfen

Andere Tätigkeit

English:

What was this OTHER activity?

Talking on the phone

Cooking/chores

Riding in a car

Childcare/helping children

Something else

If “Something else” was selected:

Haben / Sind Sie …?

gelegen

gesessen

gestanden

gegangen

gejoggt / gerannt

Were you …?

laying

sitting

standing

walking

jogging/running

Waren Sie alleine?

ja

nein

English:

Were you alone?

Yes

No

If “No” was selected:

Mit wem waren Sie zusammen?

Partner / Partnerin

Kind(er)

Andere Familienmitglieder

Freund(e)/in(nen)

Arbeitskollege(n)/Arbeitskollegin(nen)

Andere Bekannte

Person(en), die ich nicht kenne

Who were you with?

Spouse

Child(ren)

Other family members

Friend(s)

Coworkers

Other type of acquaintances

People I did not know

Wo waren Sie, bevor das Signal zur Abfrage ertönte?

Zuhause (drinnen)

Zuhause (draußen)

Auf der Arbeit (drinnen)

Draußen (nicht Zuhause)

Auto / Transporter / LKW

Anderer Ort

English:

WHERE were you just before the beep went off?

Home (indoors)

Home (outdoors)

Work (indoors)

Outdoors (not at home)

Car/van/truck

Other

If “outdoors (not at home)” was selected:

Wo waren Sie draußen? Bitte tippen Sie Ihre Antwort ein.

___________________________________________________________

WHERE were you OUTDOORS just before the beep went off? Please type in your answer.

___________________________________________________________

If “Other” was selected:

Wo waren Sie? Bitte tippen Sie Ihre Antwort ein.

___________________________________________________________

Where were you? Please type in your answer.

___________________________________________________________

**Mood (MDBF)**

Affect is assessed using an EMA version of the Multidimensional Mood State Questionnaire (Mehrdimensionaler Befindlichkeitsfragebogen [2]). The author of this version provided us with an improved revision of the German questionnaire. Participants are instructed to rate their mood in the moment “right before the prompt”. McDonald’s Omegas for the scale valence are 0.829 (within) and 0.983 (between). The scale calmness shows McDonald’s Omegas of 0.772 (within) and 0.970 (between).

*The revised version measures the three scales “valence”, “calmness” and “energetic arousal” with 8 instead of 6 bipolar items. One item was added to the valence scale (gut – schlecht; English: good – bad) and one to the calmness scale (aufgeregt – gelassen; English: aroused – composed) to increase reliability of these scales when measuring situational change. The answer format was changed from a 7-point scale to an 8-point scale, since the “neutral” response option was excluded and the scale endpoints “extreme” were added. This was done as the previous endpoints “very” were chosen extensively resulting in a negative skew of the scales valence and calmness. The adjective “energiegeladen” (English: full of energy) was exchanged for the more commonly used term “energievoll”.*

Wie fühlen Sie sich jetzt im Moment? (Moment vor Beginn der aktuellen Befragung, nicht der Moment der Befragung selbst.)

How do you feel right now? (Moment right before the prompt, not moment of prompt itself.)

Unwohl Wohl

| Extrem | Sehr | Ziemlich | Eher | Eher | Ziemlich | Sehr | Extrem |
| --- | --- | --- | --- | --- | --- | --- | --- |

Unwell Well

| Extremely | Very | Quite | Rather | Rather | Quite | Very | Extreme |
| --- | --- | --- | --- | --- | --- | --- | --- |

Entspannt Angespannt

| Extrem | Sehr | Ziemlich | Eher | Eher | Ziemlich | Sehr | Extrem |
| --- | --- | --- | --- | --- | --- | --- | --- |

Relaxed Tense

| Extremely | Very | Quite | Rather | Rather | Quite | Very | Extreme |
| --- | --- | --- | --- | --- | --- | --- | --- |

Müde Wach

| Extrem | Sehr | Ziemlich | Eher | Eher | Ziemlich | Sehr | Extrem |
| --- | --- | --- | --- | --- | --- | --- | --- |

Tired Awake

| Extremely | Very | Quite | Rather | Rather | Quite | Very | Extreme |
| --- | --- | --- | --- | --- | --- | --- | --- |

Zufrieden Unzufrieden

| Extrem | Sehr | Ziemlich | Eher | Eher | Ziemlich | Sehr | Extrem |
| --- | --- | --- | --- | --- | --- | --- | --- |

Content Discontent

| Extremely | Very | Quite | Rather | Rather | Quite | Very | Extreme |
| --- | --- | --- | --- | --- | --- | --- | --- |

Unruhig Ruhig

| Extrem | Sehr | Ziemlich | Eher | Eher | Ziemlich | Sehr | Extrem |
| --- | --- | --- | --- | --- | --- | --- | --- |

Agitated Calm

| Extremely | Very | Quite | Rather | Rather | Quite | Very | Extreme |
| --- | --- | --- | --- | --- | --- | --- | --- |

Energiegeladen Energielos

| Extrem | Sehr | Ziemlich | Eher | Eher | Ziemlich | Sehr | Extrem |
| --- | --- | --- | --- | --- | --- | --- | --- |

Full of energy Without energy

| Extremely | Very | Quite | Rather | Rather | Quite | Very | Extreme |
| --- | --- | --- | --- | --- | --- | --- | --- |

Aufgeregt Gelassen

| Extrem | Sehr | Ziemlich | Eher | Eher | Ziemlich | Sehr | Extrem |
| --- | --- | --- | --- | --- | --- | --- | --- |

Aroused Composed

| Extremely | Very | Quite | Rather | Rather | Quite | Very | Extreme |
| --- | --- | --- | --- | --- | --- | --- | --- |

Gut Schlecht

| Extrem | Sehr | Ziemlich | Eher | Eher | Ziemlich | Sehr | Extrem |
| --- | --- | --- | --- | --- | --- | --- | --- |

Good Bad

| Extremely | Very | Quite | Rather | Rather | Quite | Very | Extreme |
| --- | --- | --- | --- | --- | --- | --- | --- |

**Stress**

Subjective stress since the last prompt is assessed using 3 items. The items were adapted from Reichenberger et al. [3] making 3 changes. The first item captures how stressed the participant was since the last prompt. Responses are rated on a visual analogue scale from 0% (not at all) to 100% (very stressed). The other 2 stress items from the Perceived Stress Scale (PSS [4]) assess whether the participants felt that they “could not cope with all the things they had to do” and whether they are “on top of things” on a visual analogue scale from 0 (not at all) to 100 (very much). McDonald’s Omegas for the 3 stress items are 0.658 (within) and 0.923 (between).

Participants are also asked if a stressor occurred since the last prompt. If affirmed, participants are requested to describe the stressor. An additional question assesses the changeability of the stressor and whether the participant performed an action to change it.

*Since the main outcome measure of the Eat2beNICE-APPetite-study (impulsivity) is assessed for a time interval (since the last prompt), stress is assessed accordingly and not momentary as in Reichenberger et al.* [3]*. The first item captures how stressed the participant was since the last prompt, excluding the adjective “nervous”. Responses are rated on a visual analogue scale from 0% (not at all) to 100% (very stressed). “Not” was added to the second stress item in accordance with the original Perceived Stress Scale (PSS* [4]*): “Do you feel that you could not cope with all the things you had to do?”.*

Wie gestresst waren Sie seit der letzten Abfrage?

0% überhaupt nicht gestresst 100% sehr gestresst

How stressed have you been since the last prompt?

0% not stressed at all 100% very stressed

Wie stark hatten Sie seit der letzten Abfrage das Gefühl, mit all den anstehenden Aufgaben und Problemen nicht richtig umgehen zu können?

0 gar nicht 100 sehr stark

Do you feel that you could not cope with all the things you had to do?

0 not at all 100 very much

Wie stark hatten Sie seit der letzten Abfrage das Gefühl, alles im Griff zu haben?

0 gar nicht 100 sehr stark

Do you feel that you’re on top of things?

0 not at all 100 very much

Ist seit der letzten Abfrage ein stressiges/belastendes Ereignis eingetreten? Ein stressiges/belastendes Ereignis ist jedes Ereignis, möge es noch so geringfügig sein, das negative Auswirkungen auf Sie hat.

Ja Nein

Has a stressful event happened since the last prompt? A stressful event is any event, no matter how small, that has a negative impact on you.

Yes No

If “Yes” was selected:

Bitte beschreiben Sie das stressige Ereignis:

________________________________________________________________

Please describe the stressful event:

________________________________________________________________

If “Yes” was selected:

War das stressige Ereignis durch Sie selbst veränderbar?

Nein, ich konnte es nicht verändern.

Ja, ich konnte es verändern und habe dies getan.

Ja, ich hätte es verändern können. Meine Versuche, es zu verändern, waren jedoch nicht erfolgreich.

Ja, ich hätte es verändern können, habe es jedoch nicht versucht.

Was the stressful event modifiable for you?

No, I could not modify it.

Yes, I could modify it and did so.

Yes, I could have modified it. However, my attempts to modify it weren’t successful.

Yes, I could have modified it, but did not try to.

**Impulsivity**

State impulsivity is measured with the Momentary Impulsivity Scale (MIS [5]). The MIS consists of 4 items which are rated on a 5-point scale on how well the statement describes an individual’s behavior, cognition, and experiences since the last prompt. The MIS has a McDonald’s Omega of 0.485 (within) and of 0.833 (between).

Ich habe Dinge gesagt, ohne vorher nachzudenken.

1 = nicht zutreffend; 2 = eher nicht zutreffend; 3 = teils-teils; 4 = eher zutreffend; 5 = zutreffend

“I said things without thinking”

1 = very slightly or not at all; 2 = a little; 3 = moderately; 4 = quite a bit; 5 = extremely

Ich habe mehr Geld ausgegeben als ich sollte.

1 = nicht zutreffend; 2 = eher nicht zutreffend; 3 = teils-teils; 4 = eher zutreffend; 5 = zutreffend

“I spent more money than I meant to”

1 = very slightly or not at all; 2 = a little; 3 = moderately; 4 = quite a bit; 5 = extremely

Ich war ungeduldig.

1 = nicht zutreffend; 2 = eher nicht zutreffend; 3 = teils-teils; 4 = eher zutreffend; 5 = zutreffend

“I have felt impatient”

1 = very slightly or not at all; 2 = a little; 3 = moderately; 4 = quite a bit; 5 = extremely

Ich habe eine unüberlegte Entscheidung getroffen.

1 = nicht zutreffend; 2 = eher nicht zutreffend; 3 = teils-teils; 4 = eher zutreffend; 5 = zutreffend

“I made a ‘spur of the moment’ decision“

1 = very slightly or not at all; 2 = a little; 3 = moderately; 4 = quite a bit; 5 = extremely

**Food availability**

Each prompt assesses the food availability since the last prompt on a visual analogue scale from 0 (not available at all) to 100 (easily available).

Wie leicht verfügbar war Essen für Sie seit der letzten Abfrage?

0 - gar nicht verfügbar 100 - sehr leicht verfügbar

How easily available was food for you since the last prompt?

0 – not available at all 100 – very easily available

1. Dunton GF, Liao Y, Intille S, Huh J, Leventhal A. Momentary Assessment of Contextual Influences on Affective Response During Physical Activity. Heal Psychol. 2015;34(12):1145–1153. PMID: 26053885

2. Wilhelm P, Schoebi D. Assessing mood in daily life: Structural validity, sensitivity to change, and reliability of a short-scale to measure three basic dimensions of mood. Eur J Psychol Assess. 2007;23(4):258–267.

3. Reichenberger J, Kuppens P, Liedlgruber M, Wilhelm FH, Tiefengrabner M, Ginzinger S, et al. No haste, more taste: An EMA study of the effects of stress, negative and positive emotions on eating behavior. Biol Psychol. 2018;131:54–62. PMID: 27654506

4. Cohen S, Kamarck T, Mermelstein R. A Global Measure of Perceived Stress. J Health Soc Behav. 1983;24(4):385–396. PMID: 6668417

5. Tomko RL, Solhan MB, Carpenter RW, Brown WC, Jahng S, Wood PK, et al. Measuring impulsivity in daily life: The momentary impulsivity scale. Psychol Assess. 2014;26(2):339–349. PMID: 24274047
