# Supplementary material for: Studying Microtemporal, Within-Person Processes of Diet, Physical Activity, and Related Factors Using the APPetite-Mobile-App: Feasibility, Usability, and Validation Study
Source: J Med Internet Res. 2021 Jul 5;23(7):e25850. doi: 10.2196/25850 (PMC8406112; doi:10.2196/25850)
Supplement: Multimedia Appendix 4 [file jmir_v23i7e25850_app4.docx]

**Multimedia Appendix 4.** Bland-Altman analysis of protein, fat, carbohydrate, sugar, and fiber intake.

**Protein**

The mean difference of protein intake was not normally distributed. Therefore, natural log transformation was performed, as recommended by Bland and Altman (1986) [1]. The antilogs of the limits were taken and multiplied by 100 to allow interpretation of the ln-transformed data as percentages (100%=ideal agreement).


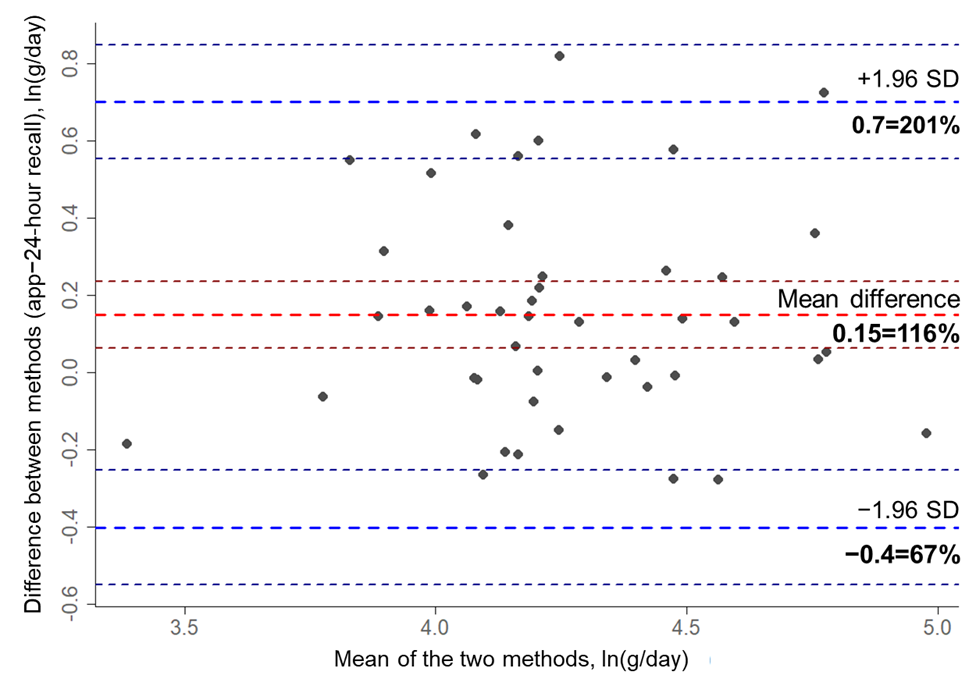


**Figure 1.** Bland-Altman plot assessing agreement between habitual protein intake in g per day captured by the APPetite-food record and the 24-hour recall (red line: mean difference=app –24-hour recall; dark red lines: 95% CI of mean difference; blue lines: lower and upper limits of agreement; dark blue lines: 95% CI of lower and upper limits of agreement).

Mean difference: 0.15=116% (95% CI 0.06 to 0.24=106.6% to 126.5%)

Lower limits of agreement: –0.4=67% (95% CI –0.55 to –0.25=57.7% to 77.6%)

Upper limits of agreement: 0.7=201% (95% CI 0.55 to 0.85=137.8% to 233.7%)

The limits of agreement transcended the predefined acceptable limits of agreement of 10% considerably.

**Fat**

The mean difference of fat intake was not normally distributed. The same procedure as described for protein intake was used.


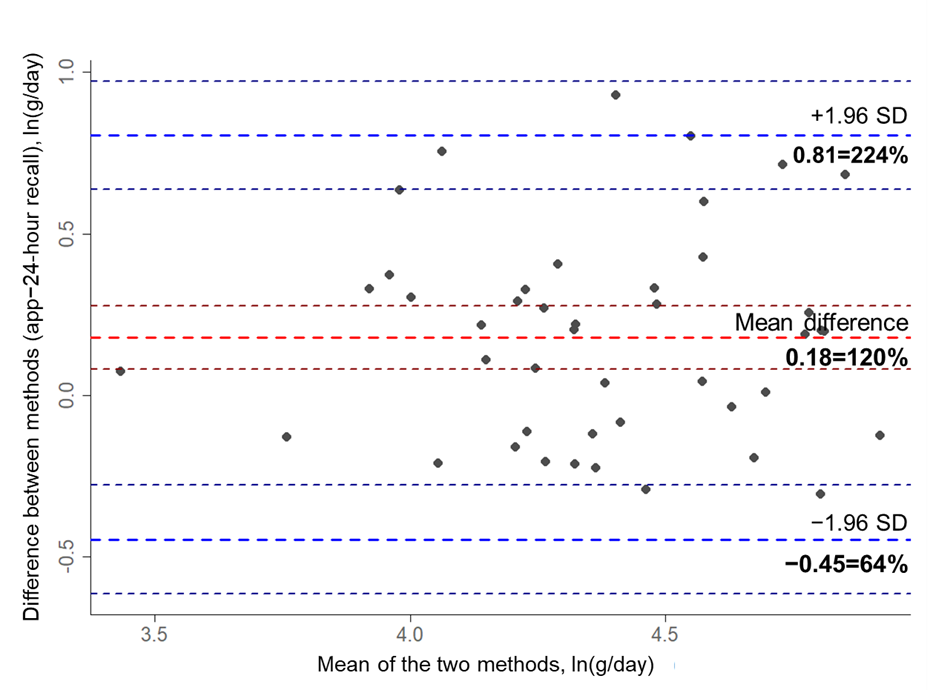


**Figure 2.** Bland-Altman plot assessing agreement between habitual fat intake in g per day captured by the APPetite-food record and the 24-hour recall (red line: mean difference=app–24-hour recall; dark red lines: 95% CI of mean difference; blue lines: lower and upper limits of agreement; dark blue lines: 95% CI of lower and upper limits of agreement).

Mean difference: 0.18=120% (95% CI 0.08 to 0.28=108.6% to 131.8%)

Lower limits of agreement: –0.45=64% (95% CI –0.61 to –0.28=54.1% to 75.7%)

Upper limits of agreement: 0.81=224% (95% CI 0.64 to 0.97=189% to 264.5%)

The limits of agreement exceeded the predefined acceptable limits of agreement of 10% substantially.

**Carbohydrates**

The mean difference of carbohydrates intake was normally distributed.


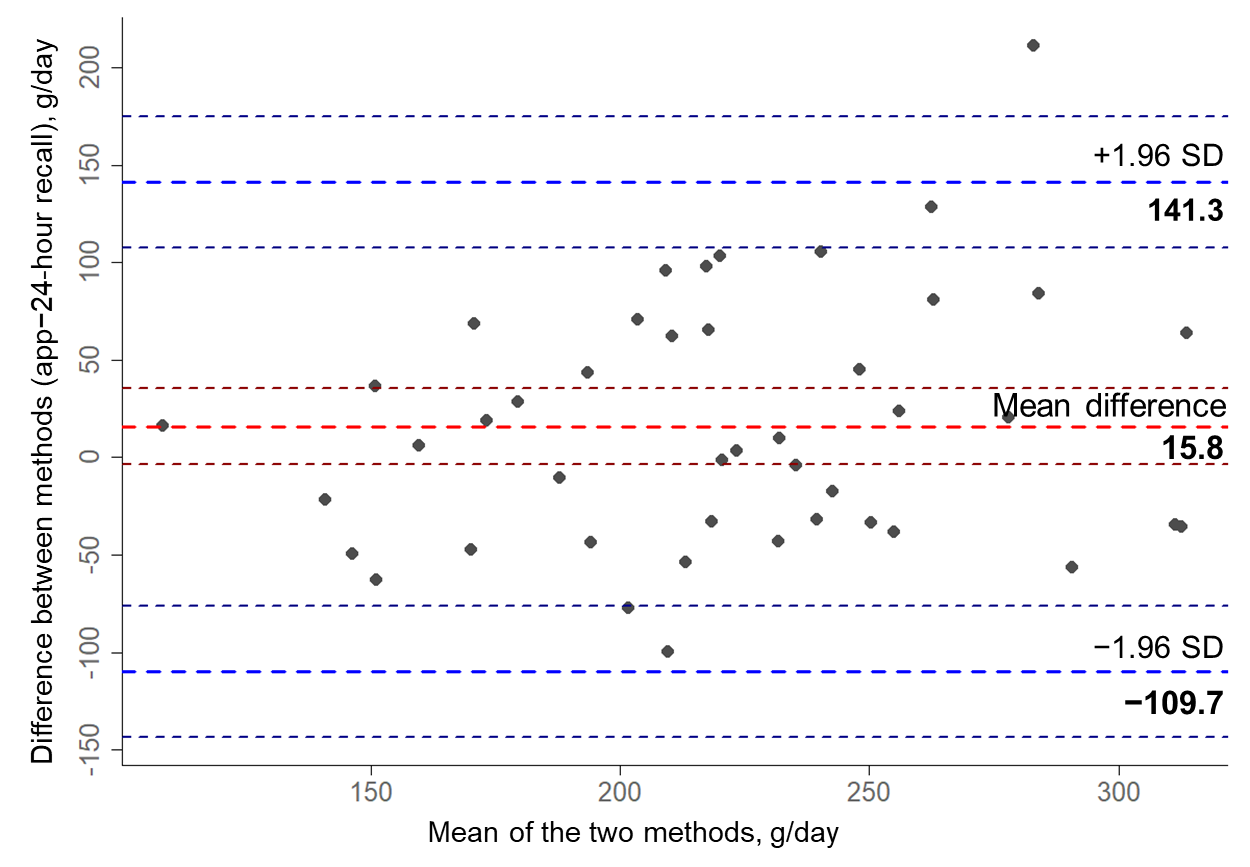


**Figure 3.** Bland-Altman plot assessing agreement between habitual carbohydrate intake in g per day captured by the APPetite-food record and the 24-hour recall (red line: mean difference=app–24-hour recall; dark red lines: 95% CI of mean difference; blue lines: lower and upper limits of agreement; dark blue lines: 95% CI of lower and upper limits of agreement).

Mean difference: 15.8 (95% CI –3.7 to 35.3)

Lower limits of agreement: –109.7 (95% CI –143.4 to –75.97)

Upper limits of agreement: 141.3 (95% CI 107.6 to 175.1)

The pre-established acceptable limits of agreement were 22.1 g, which were exceeded considerably.

**Sugar**

Normal distribution of the differences of sugar intake was given.


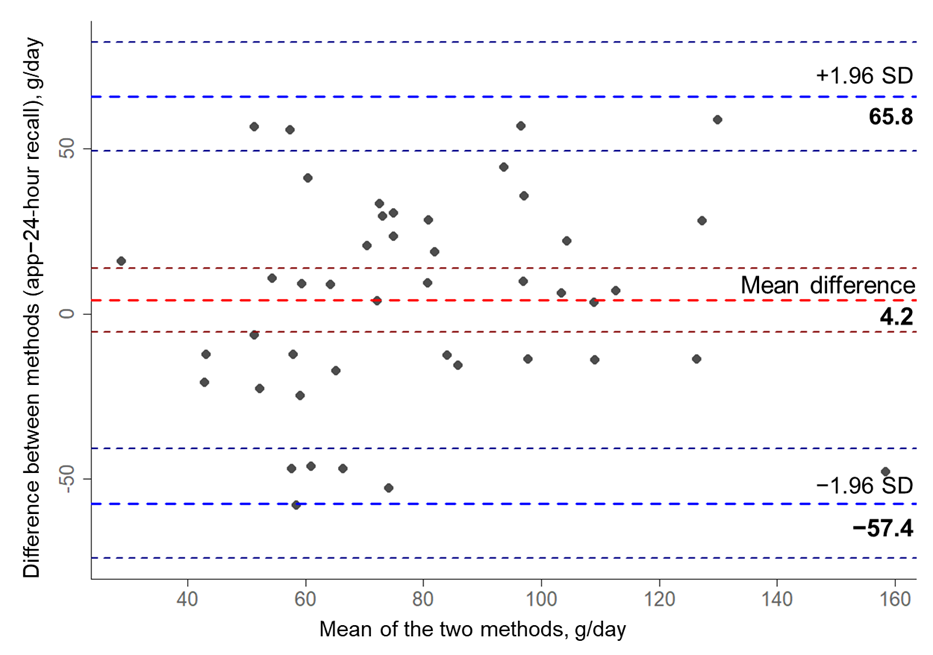


**Figure 4.** Bland-Altman plot assessing agreement between habitual sugar intake in g per day captured by the APPetite-food record and the 24-hour recall (red line: mean difference=app–24-hour recall; dark red lines: 95% CI of mean difference; blue lines: lower and upper limits of agreement; dark blue lines: 95% CI of lower and upper limits of agreement).

Mean difference: 4.2 (95% CI –5.3 to 13.8)

Lower limits of agreement: –57.4 (95% CI –73.9 to –40.8)

Upper limits of agreement: 65.8 (95% CI 49.3 to 82.4)

Disagreement of 7.9 g was predefined as acceptable, but was transcended substantially.

**Fiber**

The difference of fiber intake between the two methods was normally distributed.


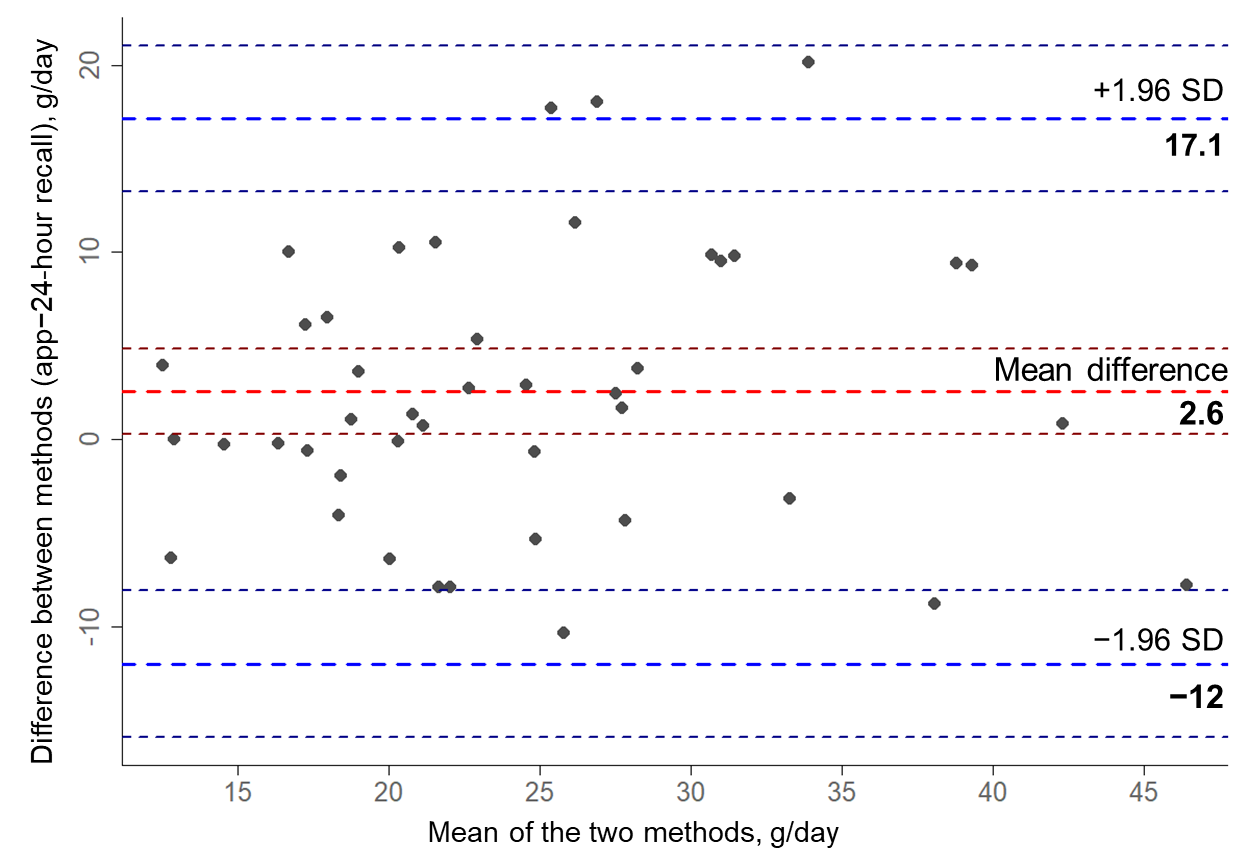


**Figure 5.** Bland-Altman plot assessing agreement between habitual fiber intake in g per day captured by the APPetite-food record and the 24-hour recall (red line: mean difference=app–24-hour recall; dark red lines: 95% CI of mean difference; blue lines: lower and upper limits of agreement; dark blue lines: 95% CI of lower and upper limits of agreement).

Mean difference: 2.6 (95% CI 0.3 to 4.8)

Lower limits of agreement: –12 (95% CI –15.9 to –8.1)

Upper limits of agreement: 17.1 (95% CI 13.2 to 21.1)

The limits of agreement exceeded the predefined acceptable disagreement of 2.5 g.

1. Bland JM, Altman DG. Statistical methods for assessing agreement between two methods of clinical measurement. Lancet. 1986;1(8476):307–310. PMID: 2868172
